# DoE-Based Optimization of a Photocatalytic C‑Alkylation Reaction in a 3D-Printed Photoreactor

**DOI:** 10.1021/acssuschemeng.5c11241

**Published:** 2026-02-16

**Authors:** Dóra Richter, Gergő Gémes, Kinga I. Hangya, Kinga Komka, Péter Kisszékelyi, Ágnes Gömöry, László Drahos, József Kupai

**Affiliations:** † Department of Organic Chemistry and Technology, Faculty of Chemical Technology and Biotechnology, 61810 Budapest University of Technology and Economics, Műegyetem rkp. 3., H-1111 Budapest, Hungary; ‡ Department of Chemical and Environmental Process Engineering, Budapest University of Technology and Economics, Műegyetem rkp. 3., H-1111 Budapest, Hungary; § Department of Organic Chemistry, Faculty of Natural Sciences, 37864 Comenius University Bratislava, Mlynská dolina, Ilkovičova 6, SK-842 15 Bratislava, Slovakia; ∥ MS Proteomics Research Group, 280964 HUN-REN Research Centre for Natural Sciences, Magyar Tudósok körútja 2, H-1117 Budapest, Hungary

**Keywords:** photoredox catalysis, 3D-printed photoreactor, 4CzIPN, radical C-alkylation, DoE optimization, energy economy factor

## Abstract

Photocatalysis provides a sustainable approach to chemical synthesis by enabling energy-efficient transformations under mild conditions. In this study, we present a comprehensive method that combines the development of photocatalytic reactions with process optimization, using a standardized 3D-printed photoreactor platform. We utilized the organophotocatalyst 4CzIPN to systematically optimize the visible-light-mediated carbon–carbon bond formation between the CH-acidic methyl cyanoacetate and 1,1-diphenylethylene. This optimization was performed through a 2^4^ full factorial design of experiments (DoE) resulting in a 48% improvement in yield (up to 91%). To address the persistent challenge of reproducibility in photochemical research, we employed a custom-designed, 3D-printed, open-access photoreactor. This design allows for standardized conditions and enhanced process optimization. We conducted a systematic investigation of various reaction parameters, including the conditions and the substrate scope. To assess the sustainability of the process, we introduced a modified energy economy factor specifically tailored for photoreactions, which illustrates a significant increase in energy efficiency. Finally, we demonstrate the possible implementation of the optimized reaction in continuous flow synthesis, underscoring the practical applicability of this methodology for advancing the design of scalable and sustainable photochemical manufacturing.

## Graphical abstract

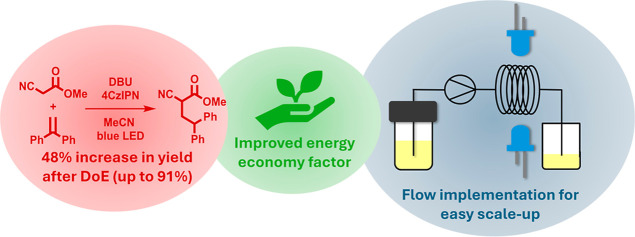

## 1 Introduction

The field of photocatalysis has experienced significant advancements over the past decade, primarily due to its numerous synthetic advantages.^1,2^ The use of visible light is especially convenient and aligns with sustainability goals by enabling chemical transformations to occur under mild conditions. The application of transition metals in redox photocatalysis, such as ruthenium and iridium polypyridyl complexes, has made this field increasingly relevant to modern synthetic objectives.^3–5^ Simultaneously, organic dyes offer a more cost-effective and easily adjustable catalytic system.^6–10^

4CzIPN is a well-known redox organo-photocatalyst that has demonstrated success in various C–C bond formation reactions (Figure 1A).^11–13^ It consists of four carbazolyl groups that act as electron donors and a dicyanobenzene central unit functioning as an electron acceptor. C–C bond formation reactions are fundamental to organic synthesis, but they can often be challenging and lack atom efficiency.^12^ A practical solution to this issue is the coupling of nucleophilic carbanions to alkenes, particularly when the carbanions are generated from readily available precursors through a photocatalytic method. Malonates and other CH-acidic derivatives can easily be converted into desired target molecules, making them an attractive group of precursors. In a prior study by Baś et al., dimethyl malonate was reacted with 1,1-diphenylethylene to synthesize such target molecules.^14^

**Figure 1:**
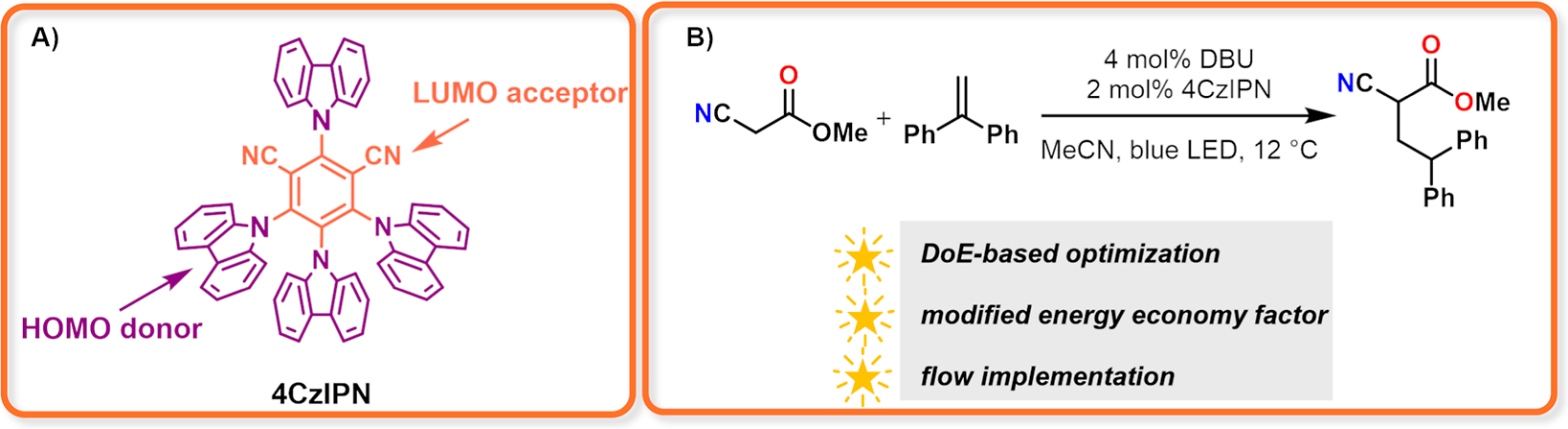
(A) Structure of 4CzIPN, (B) this work: photocatalyzed C–C bond forming reaction of the CH-acidic methyl cyanoacetate with 1,1-diphenylethylene.

The application of photocatalysis has been spreading, but reproducing these reactions can be challenging.^15–17^ The reason for this is the difficulty in accurately describing photocatalytic setups, which is especially important as the physical parameters have major influence over the outcome of the reactions. These include the type of light source, the distance between the reaction mixture and the light source, temperature control, and the intensity of mixing. Since there is no standardized setup for conducting photocatalytic reactions, researchers can choose between commercially available reactors and homemade systems. While the first option offers the potential for necessary standardization and easier transferability of reactions, homemade setups are found more commonly in the literature.^14,18–20^ This leads to the problem of inconsistent setup descriptions causing difficulties in reproducibility. To improve the comparability of experimental results for light–driven reactions, various experimental platforms have been suggested recently. We propose that the use of 3D-printed reactors can provide an affordable and easily accessible solution to these challenges. For this reason, our research was conducted using an open-access 3D-printed reactor designed by Schiel et al. that can be easily reproduced by other groups as well.^21^

To emphasize the advantages that a robust photocatalytic setup can provide, it is essential to consider the possibilities for optimization. Any type of optimization is only viable if the reaction parameters can be reliably reproduced. By conducting the reactions in a 3D-printed reactor, these conditions can be satisfied.^22^ Moreover, the optimized conditions can be applied by others using the same reactor as well. Conducting new research in this manner also complies with FAIR guidelines, which have been suggested by Wilkinson et al. in 2020 to improve findability, accessibility, interoperability, and reusability of scientific data.^23^

Other than reproducibility in photochemical setups, parameter optimization is also important for the successful realization of photocatalytic transformations. Two primary optimization techniques for chemical reactions are Bayesian optimization and design of experiments. Both methods are powerful tools, and the choice between them often depends on the specific nature of the problem at hand. Bayesian optimization requires fewer experiments than DoE, but it typically calls for expert knowledge to select appropriate parameters and can be computationally expensive. In contrast, DoE is beneficial when there are multiple input factors, but it can require a large number of experiments and may be challenging to apply in cases involving nonlinear functions. While Bayesian optimization has been used for photochemical reactions in the literature,^24^ DoE remains relatively unexplored in this field.

In this study, we integrate photocatalysis with advanced reactor engineering and systematic process optimization. Our objective was to optimize a photocatalytic process by selecting the most suitable reactant and determining the optimal reaction conditions while keeping sustainability in mind at every stage. For this reason, we expanded the substrate scope of a carbon–carbon bond formation reaction between a CH-acidic substrate and an alkene (Figure 1B). Our innovative approach of conducting DoE optimization for reaction conditions represents a novel effort in photoredox catalysis, to the best of our knowledge. This approach can enhance our understanding of such reactions. Photocatalytic reactions involve numerous parameters that influence the outcome, and identifying the statistically relevant ones can significantly improve efficiency and sustainability. It also enables us to uncover potential interactions between factors, which are often overlooked in other types of optimization processes. Moreover, we propose the introduction of a new energy economy factor that allows for meaningful comparisons of photochemical reactions from a sustainability perspective. The irradiation of reactions generates extra heat which makes the cooling of most photochemical reactions necessary. Since the current energy economy factor is not able to accurately capture the temperature difference that the cooling needs to cover, it cannot give a good basis for comparing the efficiency of different reactions. Our aim was to investigate the impact of optimization not only on the reaction yield but also on its sustainability metrics. Additionally, the optimized protocol was successfully transferred to a continuous flow system, confirming its operational stability. This transition indicates future opportunities for further improvements both in sustainability and the possibility of scale-up.

## 2 Materials and Methods

### 2.1 General Information

The starting materials and reagents were purchased from commercially available sources (Merck, TCI Europe, and VWR). Thin-layer chromatography (TLC) was performed using silica gel 60 F_254_ (Merck) plates. The spots of the materials on TLC plates were visualized by UV light at 254 nm. The reactions were monitored by TLC and high-performance liquid chromatography–mass spectrometry (HPLC–MS). The solvent ratios of the eluents are given in volume units (mL mL^–1^).

Nuclear magnetic resonance (NMR) spectra were recorded on a Bruker DRX-500 Avance spectrometer (at 500 and 126 MHz for the ^1^H and ^13^C spectra, respectively) or on a Bruker 300 Avance spectrometer (at 300 and 75.5 MHz for the ^1^H and ^13^C spectra, respectively) at specified temperatures. High-resolution mass spectrometric measurements were performed using a Q-TOF Premier mass spectrometer (Waters Corporation, 34 Maple St, Milford, MA, USA) in positive electrospray mode.

HPLC–MS was performed on an Agilent Technologies 1200 Series—Agilent Technologies 6130 Quadrupole; column: Phenomenex Kinetex C18 2.6 μm 100A 50 × 3.00 mm; A eluent: H_2_O (1% HCOONH_4_); B eluent: MeCN (8% H_2_O, 1% HCOONH_4_); gradient: 20–100%. The high-performance liquid chromatography (HPLC) yield measurements were done using a Shimadzu 2020 (Shimadzu Corp., Japan) device equipped with an Ace Excel 3C18-AR (250 × 4.6 mm) column. The exact conditions of the HPLC measurements are further described in the Supporting Information. High-resolution MS was measured on a Bruker MicroTOF II instrument using positive electrospray ionization.

The mechanochemical synthesis was done using a Retsch MM 400 ball mill with 10 mL stainless steel jars charged with two 7 mm diameter stainless steel balls.

Pictures of our setup of the open-source 3D-printed reactor can be found in the Supporting Information Figures S1–S4. The light sources used during all reactions were two 40 W Kessil A160WE Tuna Blue LEDs. For the flow reaction, PFA tubing was wrapped around the flow insert of the reactor covering a total length of 177 cm. The inner diameter of the tube was 1.5 mm, the outer diameter was 2.4 mm. The total irradiated volume was ∼11 mL.

### 2.2 Experimental Section

#### 2.2.1 General Description for Reactions Conducted in the 3D-Printed Reactor

##### 2.2.1.1 For 4 mL Size

The CH-acidic substrate (0.240 mmol) was added into a 4 mL vial along with 1,1-diphenylethylene (26.6 μL, 0.200 mmol), 1 mol % 4CzIPN (1.3 mg, 0.002 mmol), 6 mol % corresponding base (0.012 mmol) and 3.3 mL of HPLC grade acetonitrile. The reaction mixture was deoxygenated by argon bubbling for 5 min and then irradiated at 75% intensity for 8 h. The reactor chamber was thermostated at 20 °C. After 8 h the solvent was removed in vacuo, and the products were purified by preparative thin-layer chromatography.

##### 2.2.1.2 For 1.5 mL Size (on the Upper Levels of the DoE Experiments)

Methyl cyanoacetate (11.9 μL, 0.135 mmol) was added into a 1.5 mL vial along with 1,1-diphenylethylene (15.6 μL, 0.088 mmol), 4 mol % 4CzIPN (2.8 mg, 0.004 mmol), 4 mol % DBU (0.5 μL, 0.004 mmol) and 1.5 mL of HPLC grade acetonitrile. The reaction mixture was deoxygenated by argon bubbling for 5 min and then irradiated at 75% intensity for 5 h. After 5 h, a 40 μL sample was taken from the reaction mixture for HPLC yield measurement.

Control experiments were conducted on a 4 mL scale with methyl cyanoacetate as a substrate. Application of the optimized conditions for substrates other than methyl cyanoacetate was also conducted on a 4 mL scale.

##### 2.2.1.3 Flow Setup

The flow reaction was conducted using the 3D-printed flow insert of the reactor. For the reaction, methyl cyanoacetate (114 μL, 1.30 mmol) was added into a 30 mL vial along with 1,1-diphenylethylene (138 μL, 77.9 mmol), 2 mol % 4CzIPN (12.3 mg, 0.156 mmol), 4 mol % DBU (4.7 μL, 0.312 mmol) and 13 mL of HPLC grade acetonitrile. The reaction mixture was deoxygenated by bubbling argon for 5 min and irradiated at 75% intensity. The flow rate was 25 μL/min, which was calculated to provide 5 h of residence time for the irradiated volume. After the reaction was completed, a 40 μL sample was taken from the reaction mixture for HPLC yield measurement.

#### 2.2.2 Description of New Compounds

##### 2.2.2.1 Dibenzyl 2-(2,2-diphenylethyl)­malonate (**7a**)

Yellowish oil. *R*_*f*_ = 0.66 (SiO_2_ TLC, toluene:heptane:acetone = 1:1:0.1). HRMS (ESI^+^): *m*/*z* [M + Na]^+^ calcd for C_31_H_28_O_4_: 487.1885; found, 487.1885.

^1^H NMR (500 MHz, CDCl_3_, 25 °C): δ 7.38–7.33 (m, 6H), 7.32–7.26 (m, 8H), 7.22–7.17 (m, 6H), 5.15 (s, 4H), 3.93 (t, *J* = 8.0 Hz, 1H), 3.39 (t, *J* = 7.2 Hz, 1H), 2.73 (t, *J* = 7.2 Hz, 2H).

^13^C NMR (126 MHz, CDCl_3_, 25 °C): δ 169.0, 143.3, 135.4, 128.6, 128.6, 128.4, 128.4, 128.2, 127.9, 126.6, 67.1, 50.3, 48.6, 34.5, 29.7.

##### 2.2.2.2 3,3-Diphenylheptane-2,6-dione (**8b**)

Colorless oil. *R*_*f*_ = 0.57 (SiO_2_ TLC, toluene:DCM:IPA = 1:1:0.05). HRMS (ESI^+^): *m*/*z* [M + Na]^+^ calcd for C_31_H_28_O_4_: 303.1361; found, 303.1362.

^1^H NMR (500 MHz, CDCl_3_, 25 °C): δ 7.40–7.34 (m, 4H), 7.33–7.26 (m, 6H), 2.60 (t, *J* = 7.9 Hz, 2H), 2.18 (t, *J* = 7.5 Hz, 2H), 2.04 (s, 3H), 2.00 (s, 3H).

^13^C NMR (126 MHz, CDCl_3_): δ 208.3, 208.1, 141.1, 129.2, 128.5, 127.2, 65.5, 39.8, 31.2, 30.0, 29.7, 27.7.

##### 2.2.2.3 Ethyl 5-Oxo-4,4-diphenylhexanoate (**8c**)

Colorless oil. *R*_*f*_ = 0.29 (SiO_2_ TLC, toluene:DCM:IPA = 1:1:0.05). HRMS (ESI^+^): *m*/*z* [M + Na]^+^ calcd for C_31_H_28_O_4_: 333.1467; found, 333.1470.

^1^H NMR (500 MHz, CDCl_3_): δ 7.40–7.36 (m, 4H), 7.34–7.27 (m, 6H), 4.08–4.02 (q, *J* = 7.2 Hz, 2H), 2.69–2.63 (m, 2H), 2.05 (s, 3H), 2.04–2.01 (m, 2H), 1.21 (t, *J* = 7.0 Hz, 3H).

^13^C NMR (126 MHz, CDCl_3_): δ 207.7, 173.4, 140.7, 129.2, 128.5, 127.3, 65.7, 60.3, 32.5, 31.9, 31.5, 30.5, 30.2, 29.4, 27.5, 22.7, 14.2, 14.1.

##### 2.2.2.4 Methyl 2-Cyano-4,4-diphenylbutanoate (**7d**)

Colorless oil. *R*_*f*_ = 0.43 (SiO_2_ TLC, heptane/EtOAc = 4:1). HRMS (ESI^+^): *m*/*z* [M + Na]^+^ calcd for C_31_H_28_O_4_: 302.1157; found, 302.1152.

^1^H NMR (500 MHz, CDCl_3_): δ 7.38–7.22 (m, 10H), 4.28–4.23 (m, 1H), 3.78 (s, 3H), 3.39–3.32 (m, 1H), 2.84–2.75 (m, 1H), 2.65–2.58 (m, 1H).

^13^C NMR (126 MHz, CDCl_3_): δ 166.5, 142.6, 141.5, 129.1, 128.8, 128.6, 127.9, 127.6, 127.3, 127.0, 116.2, 60.4, 53.6, 48.4, 36.0, 35.4.

#### 2.2.3 Experimental Design

The optimization of methyl cyanoacetate alkylation was conducted by response surface methodology with gradient method. Statistica software (TIBCO Software Inc.) was applied for data analysis at 5% significance level to calculate the regression model and perform analysis of variance (ANOVA). A 2^4^ full factorial design was applied with three center point experiments. The effects of four independent variables (i.e., the catalyst and base amount, the molar ratio of reagents and the temperature) were investigated. The dependent variable was chosen as the yield of alkylated methyl cyanoacetate.

## 3 Results and Discussion

### 3.1 Synthesis of 4CzIPN

4CzIPN was chosen as a catalyst based on its great activity in similar C–C bond forming reactions and its simple synthesis using a mechanochemical method in adherence with sustainability guidelines.^25^ Mechanochemistry is considered a more environmentally friendly approach compared to traditional synthetic methods since it requires significantly less solvent and reduces reaction times. In this process, carbazole (**1**) and tetrafluoroisophthalonitrile (**2**) are reacted in the presence of NaO^*t*^Bu. The reaction takes only 1 h and provides the catalyst with a good yield (84%) (Scheme 1).

**Scheme 1:**
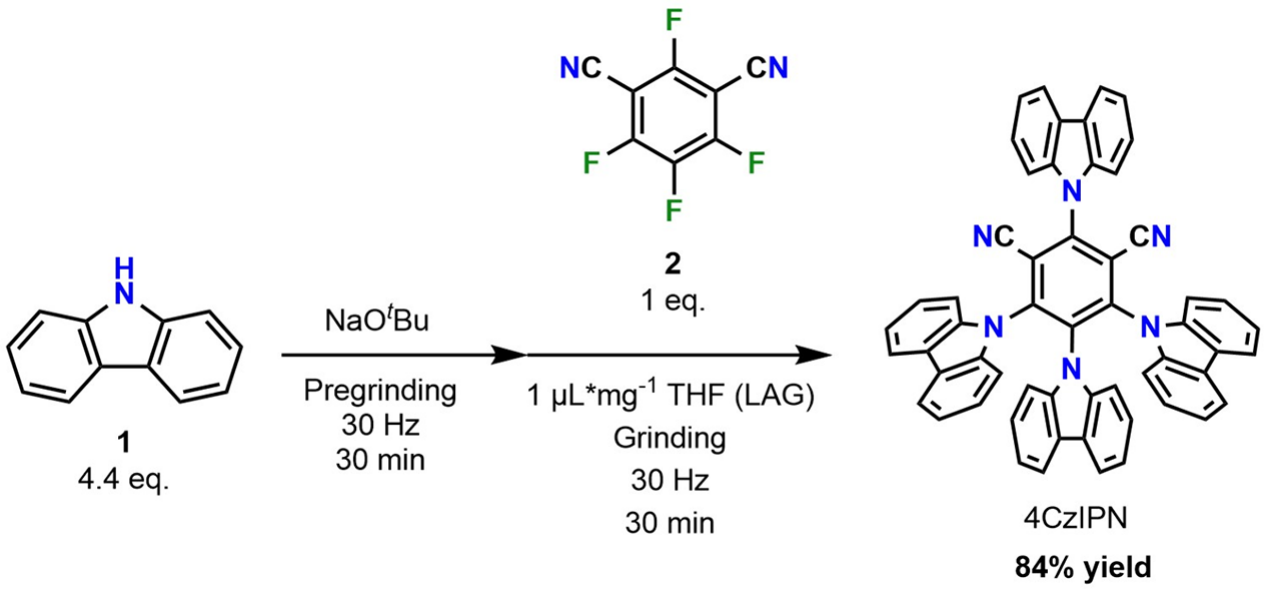
Mechanochemical Synthesis of 4CzIPN

### 3.2 Open-Access 3D-Printed Reactor

As mentioned above, having a robust setup for conducting photocatalytic reactions is a fundamental requirement. Making this setup accessible to others is the next step, enabling them to reproduce earlier results and build upon them with confidence. For this reason, we utilized an open-access 3D-printed reactor during our work, which was originally developed by Schiel and his co-workers.^21^

The reactor is designed to accommodate Kessil LED lamps and features two interchangeable light sources positioned opposite each other, offering versatility and intensity. Our construction used two 40 W Kessil A160WE Tuna Blue LEDs. The system also features vial holders of various sizes enabling rapid reaction screening for batch reactions. We used the 4 and 1.5 mL vial holders, which can accommodate 6 and 8 parallel reactions, respectively. Additionally, the design allows for a flow reaction module to be integrated into the body of the reactor, making it suitable for both batch and flow reactions. Temperature control is managed using thermoelectric coolers, which are controlled by an Arduino Nano microcontroller. For pictures of our 3D-printed photoreactor setup, refer to the Supporting Information file (Figures S1–S4).

### 3.3 C–C Bond Formation Reaction

#### 3.3.1 Investigation of the Reaction Atmosphere

We began our experiments by investigating the optimal reaction atmosphere for C–C bond forming reactions by testing the reaction between dimethyl malonate (**3**) and 1,1-diphenylethylene (**4**) (Scheme 2). To the best of our knowledge, similar studies have not been made for such reactions, and we aimed to thoroughly investigate all reaction conditions. It is well-known that oxygen can quench the excited state of photocatalysts;^26,27^ therefore removing O_2_ from the reaction atmosphere is often essential for nonoxidative reactions, although oxidative reactions can also be conducted under photoredox conditions.^28^ A common and straightforward method to achieve this is to bubble an inert gas through the reaction mixture. While this technique is convenient, it may not be the most effective way to eliminate oxygen thoroughly. To ensure maximum removal of oxygen, we applied Schlenk technique in combination with freeze–pump–thaw cycles. Additionally, we included a reaction that did not undergo deoxygenation in order to obtain a comprehensive understanding of the reaction’s behavior.

**Scheme 2:**
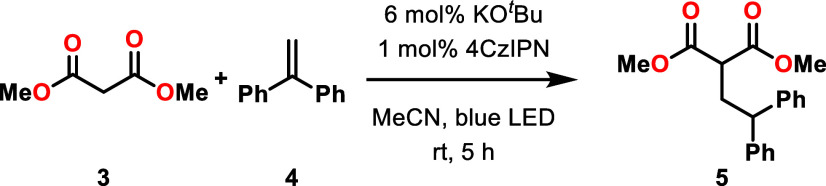
C–C Bond Forming Reaction between Dimethyl Malonate (**3**) and 1,1-Diphenylethylene (**4**)

The proposed mechanism of the C–C formation reaction is shown in Scheme 3. Following the deprotonation of the C–H acidic starting material (step I), the negatively charged **Int1** is oxidized by the photocatalyst and forms the radical **Int2** (step II). This radical then reacts with alkene **4** which gives radical **Int3** (step III). Consequently, radical **Int3** can be reduced by the photocatalyst which therefore closes the photocatalytic cycle (step IV) and concurrently forms anion **Int4** which is then neutralized by an acido–basic reaction providing the C–C coupling product (step V).

**Scheme 3:**
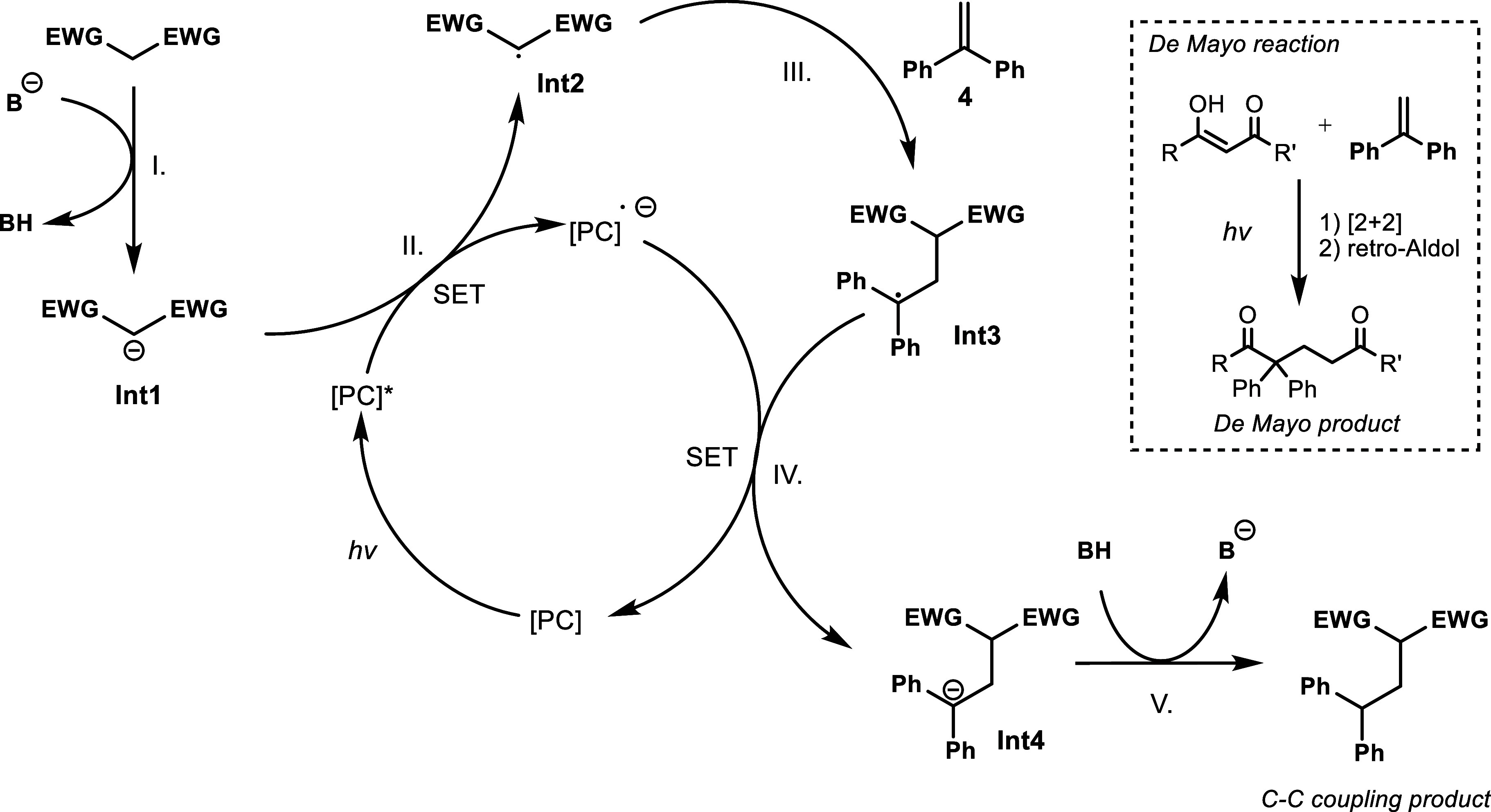
Proposed Mechanism of the C–C Bond Formation Reaction and the Potential Side-Reaction Giving the De Mayo Product^a^

a PC: photocatalyst, SET: single electron transfer, EWG: electron withdrawing group, B: general base.

As shown in Table 1, the optimal conditions are achieved through mild deoxygenation. The low yield observed under air is likely due to excessive oxygen content, which can inhibit the catalyst’s activity. However, while too much oxygen can be detrimental, it can also play a beneficial role by assisting the unreacted excited-state catalyst to return to its ground state, thus completing the catalytic cycle.^29^ Therefore, having some oxygen present can be advantageous when using Ar bubbling.

**Table 1:** Yields of Reactions Conducted under Different Atmospheres

entry	reaction conditions	yield [%]^a^
1	Ar bubbling	90
2	Schlenk + freeze–pump–thaw	14
3	air	24

a Isolated yields.

#### 3.3.2 Investigation of Other CH-Acidic Substrates

The C–C bond forming reaction involving malonates and alkenes has been extensively studied, particularly from the perspective of the alkenes.^14^ However, when it comes to converting the resulting products into desired target molecules, the functional groups of the CH-acidic substrates play a crucial role. Malonates are ideal starting materials, because the transformation possibilities of the ester functional groups are wide. In addition to the ester group, oxo and nitrile compounds can serve as excellent starting materials for such transformations as well. For nitrile compounds, one significant application of the products from the C–C bond formation reaction is their conversion into substituted β amino acids.

In our study, we examined five additional CH-acidic substrates (**6a**–**e**) illustrated in Scheme 4. The substrates were selected due to their structural similarities to the original dimethyl malonate and the potential for various transformations of their functional groups. It is important to note that for 1,3-dicarbonyl compounds, a De Mayo reaction may also occur under our reaction conditions.^30^ As seen in Scheme 3, 1,3-dicarbonyl compounds can enter into a [2 + 2] cycloaddition with the 1,1-diphenylethylene followed by ring opening through a retro-aldol reaction, creating De Mayo products.

**Scheme 4:**
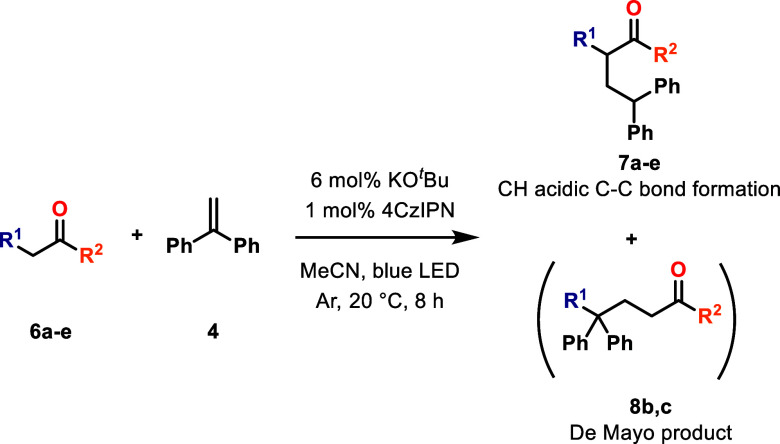
C–C Bond Forming Reactions with New CH-Acidic Substrates **6a**–**e**

The results demonstrated a wide range of yields, with dibenzyl malonate (**6a**) and ethyl cyanoacetate (**6e**) producing the best outcomes for CH-acidic alkylations (Table 2.). For acetylacetone (**6b**) and ethyl acetoacetate (**6c**), only the De Mayo product was observed. Among the new substrates, methyl cyanoacetate (**6d**) was selected for extensive optimization due to its potential to yield intermediates for substituted β amino acids.

**Table 2:** Results of Reactions between Diphenylethylene and CH-Acidic Substrates **6a**–**e**

entry	substrate	R^1^	R^2^	product	yield [%]^a^
1	**6a**	COOBn	OBn	C–C coupled (**7a**)	59
2	**6b**	COPh	Ph	De Mayo (**8b**)	40
3	**6c**	COMe	OEt	De Mayo (**8c**)	74
4	**6d**	CN	OMe	C–C coupled (**7d**)	43
5	**6e**	CN	OEt	C–C coupled (**7e**)	60

a Isolated yields.

#### 3.3.3 Control Experiments

To examine whether our reactions proceed through a photocatalytic pathway, we conducted control experiments using methyl cyanoacetate (**6d**). These reactions were performed under light irradiation without any photocatalyst, to rule out the possibility of alternative photocatalytic reactions. Additionally, to eliminate the chance of thermally activated reactions, experiments were carried out in complete darkness while using 4CzIPN as well (Scheme 5.). As no transformation of the starting materials was observed in any of these cases, we concluded that the reactions indeed proceed through a photocatalytic pathway.

**Scheme 5:**
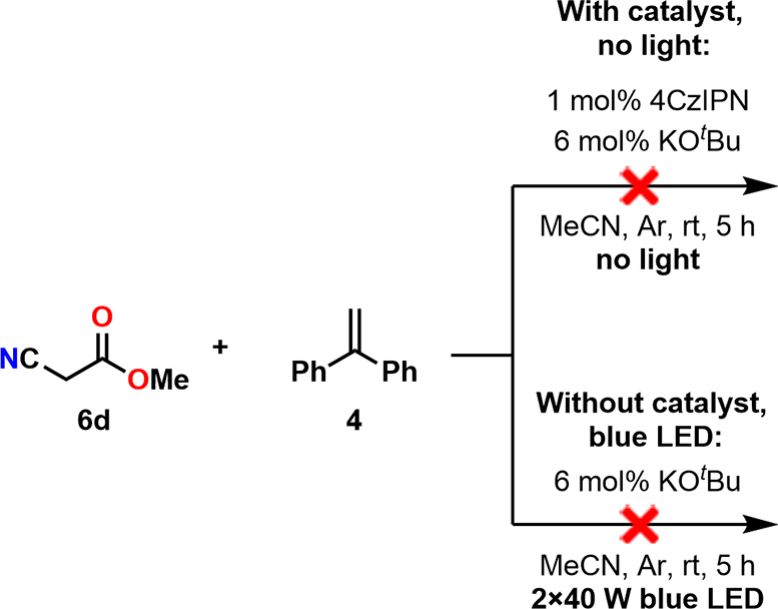
Control Experiments for Examining the Photocatalytic Pathway

### 3.4 Preliminary Studies for Experimental Design

Our plan was to optimize the yield of the reaction between methyl cyanoacetate (**6d**) and 1,1-diphenylethylene (**4**) using design of experiments. This would take advantage of the precisely adjustable parameters provided by the easily reproducible 3D-printed reactor while also using the strengths of the optimization process to expand our knowledge about the effects of the different parameters. To reliably carry out this process we had to make sure that the positions of the vials inside the reactor had no effect on the outcome of the reactions. To ensure this we conducted 8 parallel reactions where the dispersion of the yields was 1.4% (Supporting Information file Figure S24). This was a suitably low value for organic chemistry reactions; therefore, we could trust the stability of our system.

The initial parameters included the type of solvent and base, light intensity, reaction time, catalyst and base amount, molar ratio of reagents and temperature. Although the experimental design can be also applied to qualitative factors, due to the large number of factors, we decided to carry out an initial screening to identify the most effective options for the solvent and the base. The reaction conditions for this screening were the same as those used during the substrate screening to ensure comparable results (Table 3.). Among the solvents tested, MeCN had the best performance (72%, Table 3. Entry 1), which aligns with findings from other 4CzIPN-catalyzed reactions. While acetonitrile is certainly not considered a sustainable solvent, its performance is much better than any of the other examined solvents, therefore it remained our choice for the optimization. For the bases, DBU provided the highest yield (88%, Table 3. Entry 17). Additionally, using DBU helps the execution of a flow reaction due to the homogeneous reaction medium, in contrast to the heterogeneous conditions encountered when using KO^*t*^Bu.

**Table 3:** Results of the Initial Parameter Screening for Solvents and Bases

entry	solvents^a^	bases	yield [%]^b^
1	MeCN	KO^*t*^Bu	72.4
2	n-amylOAc	KO^*t*^Bu	0
3	DMC	KO^*t*^Bu	48.9
4	H_2_O + 2%DMSO	KO^*t*^Bu	1.7
5	MeSesamol^31^	KO^*t*^Bu	0
6	iBuOAc	KO^*t*^Bu	0
7	EtOH	KO^*t*^Bu	0
8	acetone	KO^*t*^Bu	24.9
9	2-Me-THF	KO^*t*^Bu	22.4
10	butanol	KO^*t*^Bu	28.2
11	^*t*^Bu-Me ether	KO^*t*^Bu	0
12	CPME	KO^*t*^Bu	6.1
13	heptane	KO^*t*^Bu	13.6
14	toluene	KO^*t*^Bu	0
15	MeCN	KO^*t*^Bu	71.5
16	MeCN	NaOEt	16.0
17	MeCN	DBU	88.3
18	MeCN	DABCO	57.3
19	MeCN	TEA	4.9
20	MeCN	DIPEA	4.7
21	MeCN	KOH	64.4
22	MeCN	Cs_2_CO_3_	79.2

a 1 mol % 4CzIPN and 6 mol % KO^*t*^Bu were used.

b Determined by HPLC (c) MeCN as solvent with 1 mol % 4CzIPN and 6 mol % base.

The remaining 6 quantitative parameters are the light intensity, the reaction time, the catalyst and base amount, the molar ratio of reagents and the temperature. Since a full factorial design would require 2^6^ reactions, a fractional design would have been necessary to reduce this number. However, this reduction would also result in a loss of information about the model due to the confounding of the effects and the interactions of the factors. Given the expected complexity of the Brønsted base—photoredox catalytic system, we believed it was more beneficial to collect as much data as possible. Therefore, two parameters, namely the light intensity and the reaction time, were also fixed prior to the execution of the DoE based on preliminary studies. With this adjustment, a 2^4^ full factorial design (including 3 center point experiments) could be implemented, which provided us with the maximum amount of data about the model while keeping the number of experiments manageable.

The light intensity was fixed at 75% based on our preliminary studies (Supporting Information Table S1). With the 75% light intensity setting, the temperature inside the reactor could be reduced to 8 °C, potentially increasing the yield of the reaction. If the light intensity was higher, the effect of the temperature change would be less exploitable due to the additional heat generated by the increased light intensity. The reaction time was determined to be 5 h based on our preliminary studies involving two parallel reactions. After 5 h, the yields did not improve significantly, leading us to conclude that this was the ideal reaction time (Figure 2).

**Figure 2:**
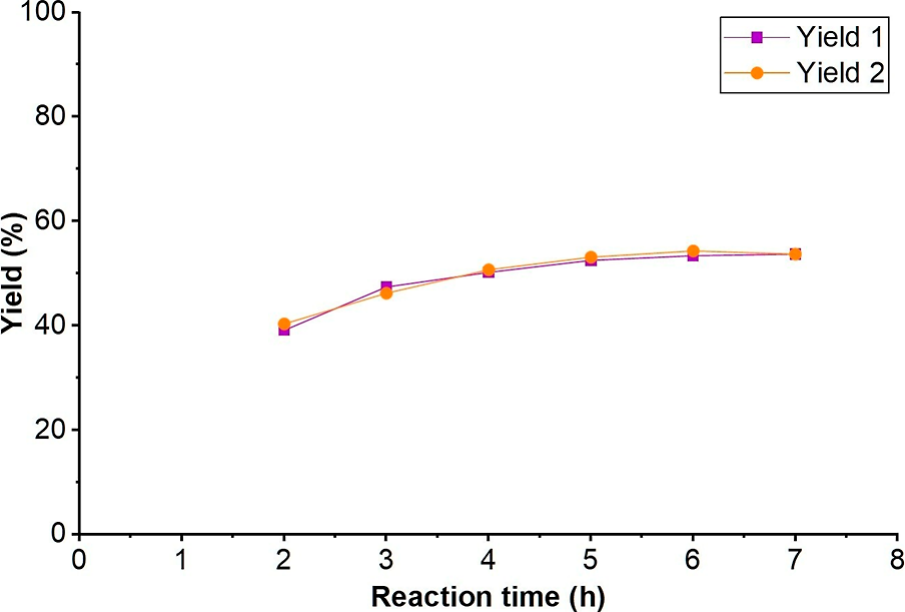
Results of the initial parameter screening for the determination of the reaction time (where Yield 1 and Yield 2 are the results of the two sets of parallel reactions). Reaction conditions: MeCN solvent, 1 mol % 4CzIPN, 6 mol % KO^*t*^Bu, 1.2 eq. methyl cyanoacetate, 20 °C.

### 3.5 Regression Model Based on Experimental Design

For the determination of the effects of the remaining four independent factors (catalyst and base amount, molar ratio of reagents and temperature), a 2^4^ full factorial design was performed with three center point experiments. The dependent variable, as previously defined, was set as the nonisolated alkylated methyl cyanoacetate (**7d**) yield and was modeled as a function of the independent parameters using linear regression. The upper and lower levels of the factors were also determined based on preliminary studies that changed each parameter individually (Supporting Information, Figure S25). Regarding the reaction temperature, the results of former experiments show that lower temperatures would yield better results, however, we also wanted to take energy efficiency into account. Without cooling, the reactor’s internal temperature rises to 44 °C. Conducting reactions at higher temperatures would require less cooling and therefore consume less energy. For this reason, we aimed to investigate the effects of temperature above room temperature as well. Molar ratio is defined as the ratio of methyl cyanoacetate (**6d**) to 1,1-diphenylethylene (**4**). The lower and upper values of the factors are presented in Table 4, while Table 5 displays the experimental design matrix alongside the observed alkylated methyl cyanoacetate (**7d**) yields.

**Table 4:** Levels of the Factors^a^

independent variables	factor	lower level	center point	upper level
temperature [°C]	*A*	20	30	40
base [mol %]	*B*	2	3	4
catalyst [mol %]	*C*	2	3	4
molar ratio [−]	*D*	0.75	1.125	1.5

a Molar ratio is defined as the ratio of methyl cyanoacetate (**6d**) to 1,1-diphenylethylene (**4**).

**Table 5:** Experimental Design Matrix and Observed **7d** Yields^a^

entry	temperature [°C]	base [mol %]	catalyst [mol %]	molar ratio [−]	yield [%]
1	40	4	4	1.5	58.7
2	20	4	4	1.5	82.9
3	40	2	4	1.5	54.0
4	40	4	2	1.5	63.8
5	40	4	4	0.75	42.8
6	20	2	4	1.5	74.3
7	20	4	2	1.5	78.5
8	20	4	4	0.75	62.9
9	40	2	2	1.5	54.6
10	40	2	4	0.75	35.1
11	40	4	2	0.75	42.1
12	20	2	2	1.5	90.6
13	20	2	4	0.75	56.9
14	20	4	2	0.75	60.6
15	40	2	2	0.75	33.9
16	20	2	2	0.75	70.3
17 (C)	30	3	3	1.125	59.3
18 (C)	30	3	3	1.125	58.9
19 (C)	30	3	3	1.125	57.7

a (C): Center point.

The estimated effects of the factors and the interactions are shown in Table 6. Since the curvature is not significant, the linear model is suitable. A factor is considered statistically significant if its *p*-value is less than 0.05. Additionally, a higher *t*-value indicates that a factor has a more significant impact on the model’s response. Model reduction is a process of simplifying the model by removing the nonsignificant factors, while retaining the accuracy of the model. Using a Pareto Chart (Figure 3.) for this can be a great method, since the scale of the effects can also be compared to each other. By only taking the outstandingly large effects into account, it is possible to get a sufficiently accurate model, while considerably simplifying it. Based on these considerations, only effects *A* and *D* were found to be significant in our case.

**Table 6:** Effects Estimate Table for the Linear Regression Model

factor	effect	standard error	*t*(3)	*p*-value
mean/interc	60.124	0.363	165.575	0.000
curvature	–3.037	1.828	–1.662	0.195
(A) temperature (°C)	–23.995	0.726	–33.040	0.000
(B) base (mol %)	2.816	0.726	3.878	0.030
(C) catalyst (mol %)	–3.358	0.726	–4.623	0.019
(D) molar ratio (−)	19.081	0.726	26.274	0.000
A by B	4.606	0.726	6.342	0.008
A by C	2.409	0.726	3.318	0.045
A by D	0.237	0.726	0.326	0.766
B by C	3.943	0.726	5.429	0.012
B by D	–0.206	0.726	–0.284	0.795
C by D	–1.042	0.726	–1.435	0.247
Aby B by C	–5.161	0.726	–7.106	0.006
Aby B by D	–0.272	0.726	–0.375	0.733
A by C by D	–0.843	0.726	–1.160	0.330
B by C by D	0.125	0.726	0.172	0.874

**Figure 3:**
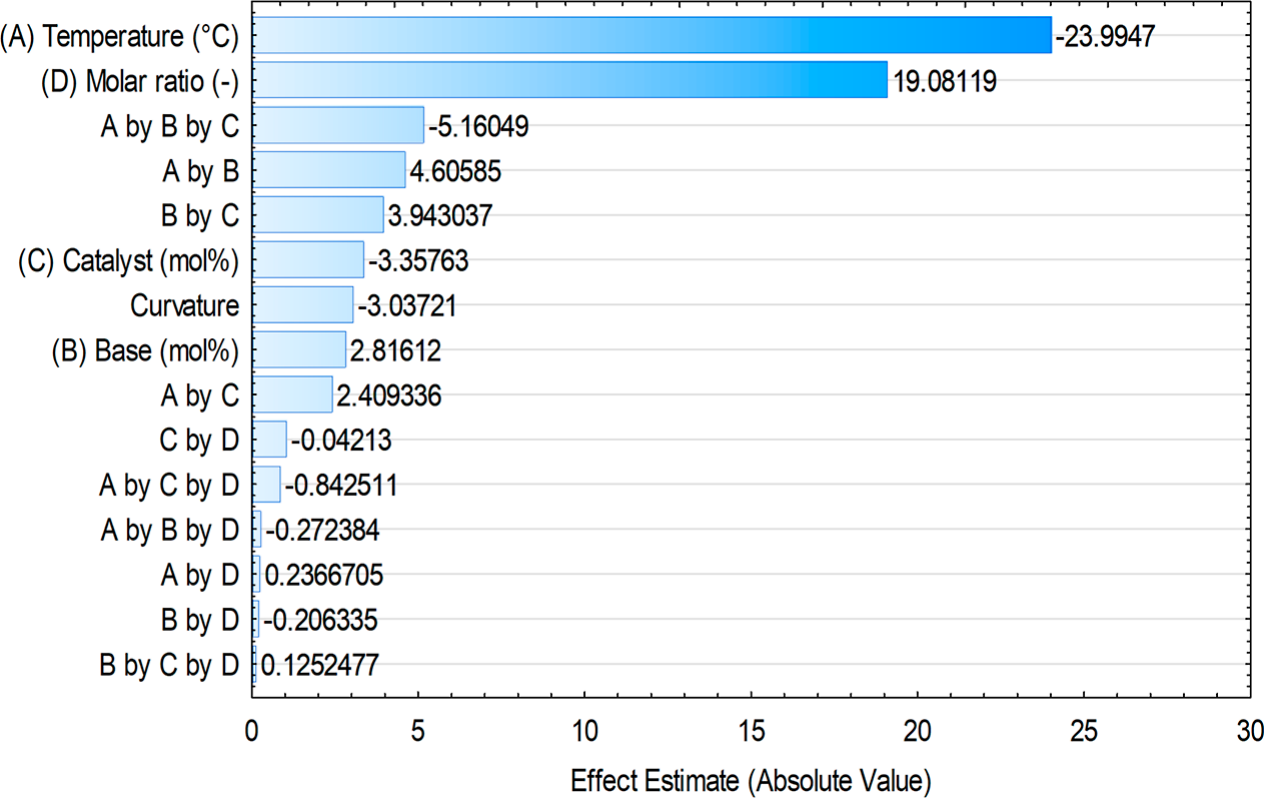
Pareto Chart of the effects in the case of yield (**7d**) as the dependent variable.

After the model reduction, a new linear regression model was constructed, containing only the significant factors. With this simple model, both a high *R* ^2^ value (0.91) and a good adjusted *R* ^2^ value (0.89) were achieved. The results for the reduced model can be seen in Table 7.

**Table 7:** Effects Estimate Table for the New Reduced Linear Regression Model

effect	effect	standard error	*t*(3)	*p*-value
mean/interc	60.124	1.238	48.563	0.000
curvature	–3.037	6.231	–0.487	0.633
(A) temperature (°C)	–23.995	2.476	–9.690	0.000
(D) molar ratio (−)	19.081	2.476	7.706	0.000

After checking the assumptions of linear regressions, namely that the variance of errors is constant (Supporting Information Figure S28), and that the errors follow a normal distribution (Supporting Information Figure S29), the adequacy of the model was also confirmed through validation experiments. Reactions were conducted simultaneously in the upper half of the design space at three different molar ratio values (Table 8). These experiments also showed good accordance with the predicted results, as both the confidence and the predicted intervals contain our results, though measured yields were consistently below the predicted values. The reason for this could be that during the model reduction some statistically significant parameters with minimal effects have also been disregarded, that could affect the predicted yield. Based on these results, the reduced model can be accepted to predict the results of the reaction with adequate accuracy and can be used for optimization purposes. The final linear regression model representing the relationship between yield (*Y*) and the coded values of the independent factors is expressed as

1 Y=60.1−12.0A+9.5D

where *A* represents the temperature (°C) and *D* represents the molar ratio of methyl cyanoacetate (**6d**) to 1,1-diphenylethylene (**4**).

**Table 8:** Observed and Predicted **7d** Yields of the Validation Experiments^a^

entry	molar ratio [−]	yield [%]	predicted yield [%]	–95% conf	+95% conf	–95% pred	+95% pred
1	1.5	77.0	81.7	77.1	86.2	70.2	93.2
2	1.5	80.8	81.7				
3	1.4	73.7	79.1	74.9	83.3	67.8	90.5
4	1.4	73.7	79.1				
5	1.3	73.7	76.6	72.6	80.5	65.38	87.8
6	1.3	72.9	76.6				

a Reaction conditions: MeCN solvent, 2 mol % 4CzIPN, 4 mol % DBU, 20 °C.

The effect of the temperature (*A*) and the molar ratio (*D*) to the yield was illustrated on a response surface plot at 4 mol % base and 2 mol % catalyst (Figure 4.). The graph shows that the yield increases linearly as temperature decreases and molar ratio increases. This information provides guidance for the subsequent optimization reactions.

**Figure 4:**
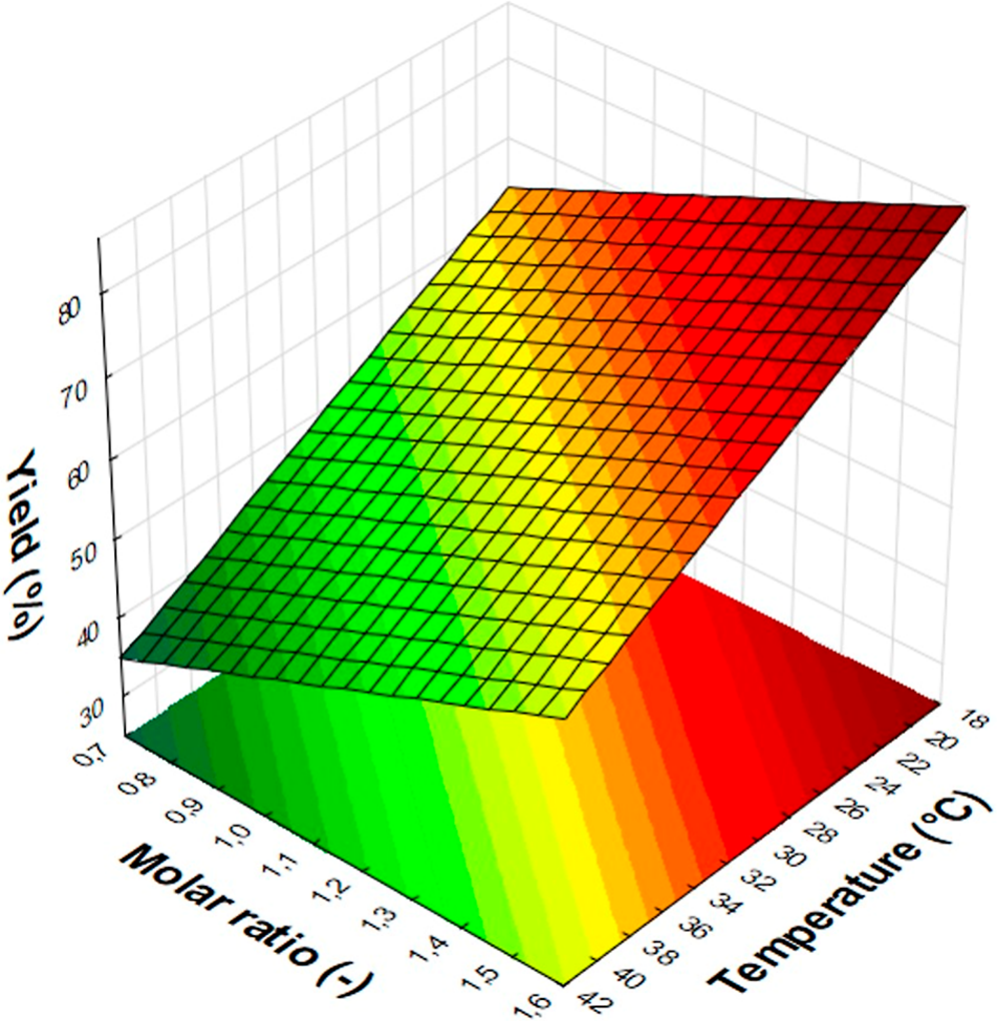
Response surface plot for the effects of the temperature (*A*) and the molar ratio (*D*) at 4 mol % base and 2 mol % catalyst.

### 3.6 Reaction Optimization

The optimization was carried out using response surface methodology combined with gradient method. In this approach, the gradient direction extends from the center point of the design space toward the local maximum of the reduced model, as this indicates the steepest ascent. The direction of this steepest ascent is defined by the ratio of the two coefficients of the reduced model. Steps are taken along this gradient with intervals chosen adequately. In our study, the temperature was the leading factor since it has the most significant effect among the variables considered. Based on the former model, the temperature has a negative effect (−12*A*), so it has to be decreased to reach greater yield. Using the set light intensity of 75%, the reactor’s lowest stable temperature setting was determined to be 8 °C. The range from the lower setting of the temperature (20 °C) to the lowest achievable temperature (8 °C) was divided into three intervals, as shown in Table 9. The setting for the other parameter (*D*) can be calculated based on the gradient method and the steps chosen for the leading parameter. The two other factors (*B*, *C*) had no significant effects, so they were set at one of the settings of the former design, at 4 mol % and 2 mol %, respectively.

**Table 9:** Temperature and Calculated Molar Ratio Settings for the Gradient Method Optimization

steps	temperature [°C]	molar ratio [−]
0	30	1.125
1	20	1.423
2	17	1.513
3	12	1.662
4	8	1.781

The reduced model is defined as valid only within the design space. Consequently, when stepping out of it, it is necessary to compare the measured results to the results predicted by the model to check for adequacy. Since our model indicated possible further increase in yields, it led us to conduct reactions beyond the design space for optimization (Figure 5.). Step 0 is the center point with already known values while Step 1 is still part of the original design space, therefore no experiment is performed at that level. In Step 2, the results matched the predicted values, suggesting that yield could still be improved further. However, in the next step, the yields were lower than predicted, showing a deviation from the linear model, and starting a trend for the flattening of the curve. Step 4 continued this trend, giving only marginally better yields than the previous one and diverging further from the predicted value. Based on these results, Step 3 was accepted as the optimal reaction conditions. Lowering the temperature to 8 °C in Step 4 did not provide any significant advantages, while 12 °C in Step 3 requires less energy for cooling. Therefore, the most favorable conditions were determined to be 12 °C, a 1.66 molar ratio, 4 mol % base, and 2 mol % catalyst.

**Figure 5:**
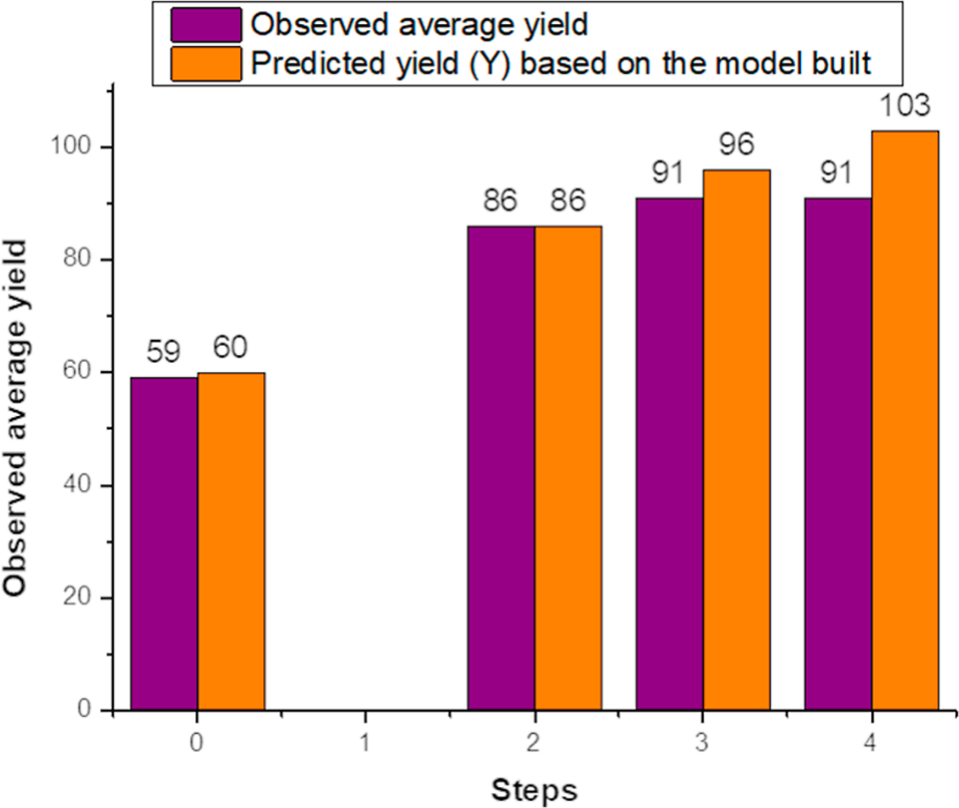
Observed and predicted **7d** yields for the optimization experiments (As Step 1 is part of the original design space, no experiment was performed at that level.).

### 3.7 Application of the Optimized Conditions for Other Substrates

After establishing the optimized conditions for the reaction of methyl cyanoacetate (**6d**) and 1,1-diphenylethylene (**4**), we aimed to determine whether these conditions are generally applicable for all C–C bond forming reactions or if they are specific to the methyl cyanoacetate. Reactions were conducted using the optimized conditions with compounds **6a**–**c** and **3**, then the results were compared with the initial yields (Table 10.).

**Table 10:** Yields of the C–C Bond Forming Reactions of Other CH-Acidic Substrates (**6a**–**c** and **3**) and Comparison with the Initial Results

entry	substrate	reaction type	yield [%]^a^	previous yield [%]^a^
1	**6a**	C–C coupling	2	59
2	**6b**	De Mayo	16	40
3	**6c**	De Mayo	traces	74
4	**3**	C–C coupling	3	90

a Isolated yields.

It was found that the results showed a significant decrease in yields when the optimized reaction conditions were applied to all substrates. Based on these findings, the optimized conditions are only suitable for methyl cyanoacetate. Separate optimizations should be conducted for other substrates as needed.

### 3.8 Preliminary Study for Conducting Flow Synthesis

Alongside traditional batch photocatalytic reactions, photoflow reactions have recently gained popularity as well.^32^ One key benefit of photoflow reactions is improved light penetration of the reaction mixture, as light is primarily absorbed by only the outer layer of the reaction mixture. Due to this, using a reaction vessel with a smaller diameter can result in better yields and shorter reaction times in photochemical applications. This makes flow chemistry an ideal approach when combined with photocatalysis. In flow chemistry, the reaction solution moves through narrow tubing, which allows for maximum light penetration. Additionally, scaling up photocatalytic reactions is easier with flow systems as they can operate without increasing the necessary irradiated volume. These design features can make the processes significantly more efficient and can increase sustainability. In our case, flow implementation would be the only feasible mode of scale up, as the batch reaction sizes are limited by the vial holders of the reactor as well.

The reaction of methyl cyanoacetate (**6d**) and 1,1-diphenylethylene (**4**) was conducted using the flow setup of our 3D-printed reactor as a preliminary study (Figure 6.). The reaction conditions matched those that were selected as the optimal batch conditions, however, flow optimization could be performed later. The established residence time was 5 h, which can most likely be significantly reduced through such optimization.

**Figure 6:**
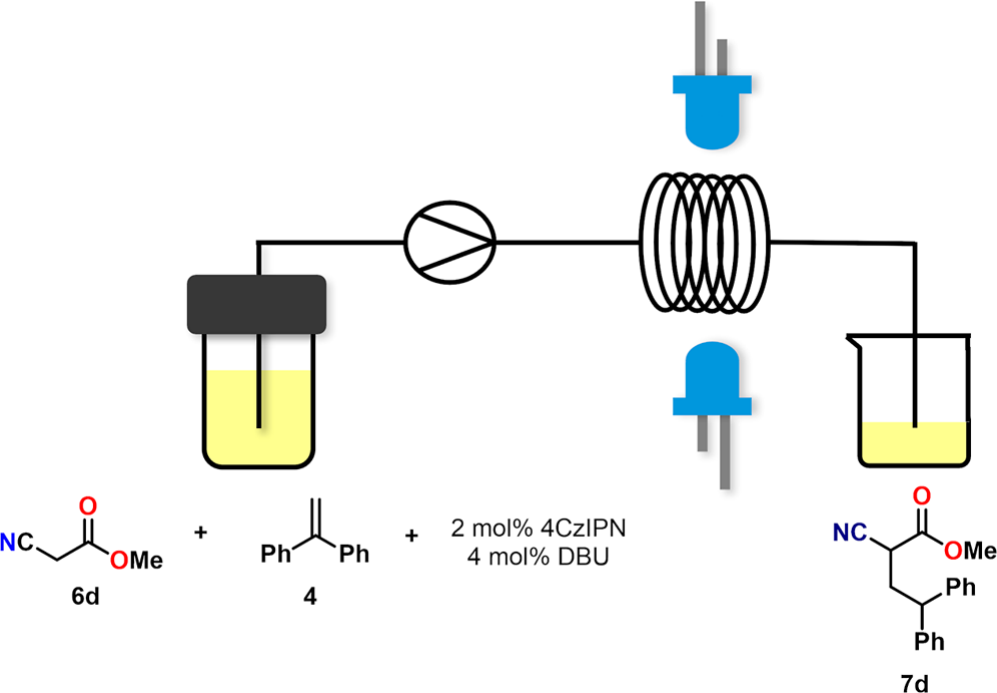
Photoflow execution of the reaction of methyl cyanoacetate (**6d**) and 1,1-diphenylethylene (**4**).

The flow reaction provided results comparable to the optimized batch reaction, achieving a yield of 91%. After flow optimization, it presents a great opportunity for scaling up the reaction.

### 3.9 Environmental Energy Impact

The energy economy factor (ε) is defined as the yield of a reaction divided by the temperature multiplied by the reaction time. This metric, which is important in green chemistry, allows for the evaluation of processes based on the effectiveness of the reaction relative to the energy input required. Both the yield and the energy input significantly influence the feasibility of the process. This metric is primarily designed for heated reactions, as heating to high temperatures can have considerable environmental impacts. While it provides meaningful comparisons for heated reactions, it overlooks ambient temperature and cooled reactions. To address this limitation, our group has contributed by proposing a corrected energy economy factor (ε_corr_) for ambient temperatures.^31^

To describe our current work more clearly, we propose further modifications to the corrected energy economy factor. The heat generated by the light sources can significantly increase the temperature inside the reactor, even with LED lights. This phenomenon makes photoreactions similar to exothermic reactions regarding energy usage, as reaching room temperature requires a considerable investment of energy. Therefore, the relevant temperature difference is not simply between the reaction temperature and room temperature, but rather between the highest temperature the system can reach and the temperature at which the reaction needs to occur. This is the actual temperature difference that represents the energy investment required. Consequently, we propose a new, modified energy economy factor (ε_photo_) that accounts for the temperature difference between *T*_reactor_ (temperature inside the reactor without cooling) and *T*_reaction_ (the reaction temperature) to accurately describe the total energy investment needed (eq 2). This way, higher ε_photo_ values represent better and more sustainable results, while lower ε_photo_ values mean less efficiency—lower yields, longer reaction times or larger temperature difference that requires cooling.

Including the internal temperature of the reactor without cooling in the energy economy factor will enhance our understanding of the energy requirements for photoreactions, potentially leading to more accurate predictions of energy use and improved process design. When *T*_reactor_ equals *T*_reaction_, we have defined ε_photo_ as the quotient of the yield (Y) and the reaction time (*t*) to avoid division by zero (eq 3.). The current limitation of this photo energy economy factor definition is that the internal temperature of reactors without cooling is not well-documented in the literature. To facilitate comparisons from an environmental impact perspective, we recommend including this data in future studies. The calculated ε_photo_ can be seen in eq 3 for our case as an example.

2 $$\varepsilon_{photo}=\frac{Y}{\left|T_{reactor}-T_{reaction}\right|\cdot t}$$

3 $$\varepsilon_{photo}=\frac{Y}{t},\text{if},T_{reactor}-T_{reaction}=0$$

*Y* = yield [−], *T* = temperature [°C], *t* = reaction time [h].

In our case

4 εphoto=0.906|44°C−12°C|·5[h]=5.66·10−3C°−1h−1

To ensure that the energy economy factors of our photocatalytic reactions can be compared with similar published reactions despite the absence of uncooled reactor temperature data, we have defined a general energy economy factor (ε_general_) (eq 5). This factor calculates the absolute difference between the reaction temperature and room temperature (set at 25 °C).^31^ While it does not account for the energy required for cooling, it offers a general indication of the scale of different reactions. Importantly, this definition is versatile; it is not restricted to cooling alone but can also be applied to heating, ambient temperature conditions, and nonexothermic cooled reactions. If the reaction temperature is equal to room temperature, ε_general_ is defined as the quotient of the yield divided by the reaction time to avoid division by zero (eq 6). A comparison of the ε_general_ values for various photocatalytic C–C bond forming reactions is presented in Table 11.

5 $$\varepsilon_{general}=\frac{Y}{\left|T_{reaction}-{25\;}^{{}^{\circ}}C\right|\cdot t}$$

6 $$\varepsilon_{general}=\frac{Y}{t},\text{if},T_{reaction}={25\;}^{{}^{\circ}}C$$

**Table 11:** General Energy Economy Factors for Photocatalytic C–C Bond Forming Reactions

reference	reaction	temperature [°C]	reaction time [h]	yield [−]	ε_general_ [°C^–1^ h^–1^]
this work	C–C Bond forming reactions of malonates with styrenes	12	5	0.906	–0.014
this work at rt	C–C Bond forming reactions of Malonates with Styrenes	25	5	0.70	0.140
 ^14^	C–C Bond forming reactions of malonates with styrenes	25	20	0.91	0.046
 ^33^	oxidative photodimerization	25	20	0.66	0.033
 ^34^	1,2-dDicarbonylation of alkenes toward 1,4-diketones	25	24	0.73	0.030
 ^35^	decarboxylative radical addition bifunctionalization cascade	25	12	0.90	0.075

The comparison of different types of photocatalytic C–C bond formations reveals that most reactions have a general energy economy factor within a small range. For the reaction discussed in this work, the ε_general_ was calculated for both the optimized conditions at 12 °C and for a reaction conducted at room temperature. Among the examples presented in Table 11, our room temperature reaction demonstrated the best energy economy factor, an order of magnitude better than most of the others. This value also highlights the difference between a reaction optimized for maximum yield, and one optimized for energy efficiency and sustainability; a lower yield can provide a better ε_general_ value. Furthermore, the problem with the definition of the temperature difference is evident, as the ε_photo_ value (eq 4) calculated for the optimized reaction conditions is 2 orders of magnitude lower than the ε_general_ at room temperature and 1 order of magnitude lower than the ε_general_ at 12 °C. This emphasizes the importance of clearly defining the photo energy economy factor.

## 4 Conclusion

In this study, we developed an integrated photocatalytic methodology that combines reaction optimization, reaction engineering, and sustainability assessment. Using 4CzIPN as an organophotocatalyst, we achieved carbon–carbon bond formation between CH-acidic substrates and 1,1-diphenylethylene under mild, photoredox conditions. During the carbon–carbon bond forming reaction, four new substrates were identified. Among these, methyl cyanoacetate was selected for extensive optimization using DoE to produce intermediates for substituted β-amino acids.

Process optimization was conducted using a 2^4^ full factorial design, which identified temperature and substrate ratio as key parameters, resulting in up to 91% product formation. The implementation of a custom 3D-printed photoreactor allowed for reproducible reaction conditions and precise control of parameters, facilitating systematic data collection and enhancing process reliability. The optimized conditions were successfully adapted to a continuous flow setup, demonstrating comparable yields to those obtained in batch reactions and demonstrating the potential for scalability. To quantitatively evaluate the sustainability of the process, we introduced a modified energy economy factor (ε_photo_). This new metric accounts for the total temperature range requiring cooling, offering a more realistic assessment of energy demands in photocatalytic systems. Under this framework, our room temperature reaction showed an order of magnitude improvement in energy efficiency compared to conventional carbon–carbon bond forming processes. Overall, this work illustrates how the integration of photocatalytic methodology, 3D-printed reactor technology, and quantitative energy assessment can advance the design of scalable and sustainable photochemical manufacturing routes.

## Supplementary Material



## Notes

Reactor setup, NMR and HRMS characterization of new materials, HPLC chromatograms, investigation of the potential effect of the reaction vial placement on the yields, preliminary studies for design of the experiments and further results of DoE (PDF)

## Abbreviations

4CzIPN: 1,2,3,5-tetrakis­(carbazol-9-yl)-4,6-dicyanobenzene
CPME: cyclopentyl methyl ether
DABCO: 1,4-diazabicyclo­[2.2.2]­octane
DBU: 1,8-diazabicyclo­[5.4.0]­undec-7-ene
DIPEA: *N*,*N*-diisopropylethylamine
LAG: liquid-assisted grinding
PFA: perfluoroalkoxy alkane
TEA: triethylamine.
