# Supplementary material for: DoE-Based Optimization of a Photocatalytic C‑Alkylation Reaction in a 3D-Printed Photoreactor
Source: ACS Sustain Chem Eng. 2026 Feb 16;14(8):3958–69. doi: 10.1021/acssuschemeng.5c11241 (PMC12958348; doi:10.1021/acssuschemeng.5c11241)
Supplement: Supplementary file 1 [file sc5c11241_si_001.pdf]

Supporting Information

DoE-based optimization of a photocatalytic C-alkylation reaction in a 3D-printed photoreactor

Dóra Richter, Gergő Gémes, Kinga I. Hangya, Kinga Komka, Péter Kisszékelyi, Ágnes Gömöry, László Drahos and József Kupai

Number of pages: 20

Number of figures: 29

Number of tables: 1

Table of contents

1. Open-access 3D-printed reactor 2

2. Characterization of new materials 4

   2.1. Dibenzyl 2-(2,2-diphenylethyl)malonate (7a): 4

   2.2. 3,3-Diphenylheptane-2,6-dione (8b): 6

   2.3. Ethyl 5-oxo-4,4-diphenylhexanoate: 8

   2.4. Methyl 2-cyano-4,4-diphenylbutanoate (7d): 10

3. HPLC yield determination for alkylated methyl cyanoacetate (7d) 12

4. Investigation of the potential effect of the reaction vial placement on the yields 16

5. Preliminary studies for Design of Experiments 17

   5.1. Base and catalyst amount screening 17

   5.2. Investigation of the effect of light intensity on the C-C bond forming reaction 17

6. Further results of Design of experiments 18

   6.1. Full effects estimate tables of the DoE models including confidence limits 18

   6.2. Checking the constant error variance assumption 19

   6.3. Checking the normal distribution of errors assumption 19

References 20

1. Open-access 3D-printed reactor

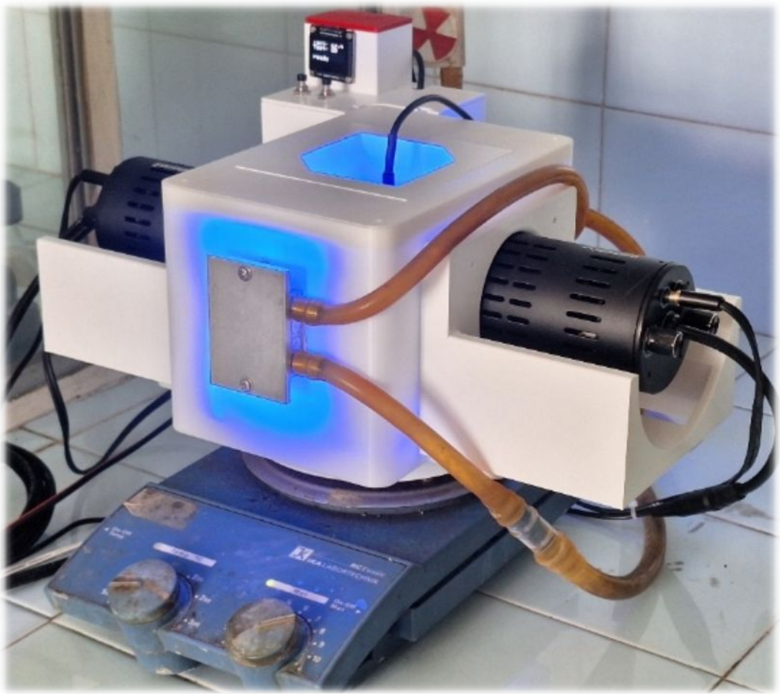

Figure S1. Picture of the outside of the reactor during operation

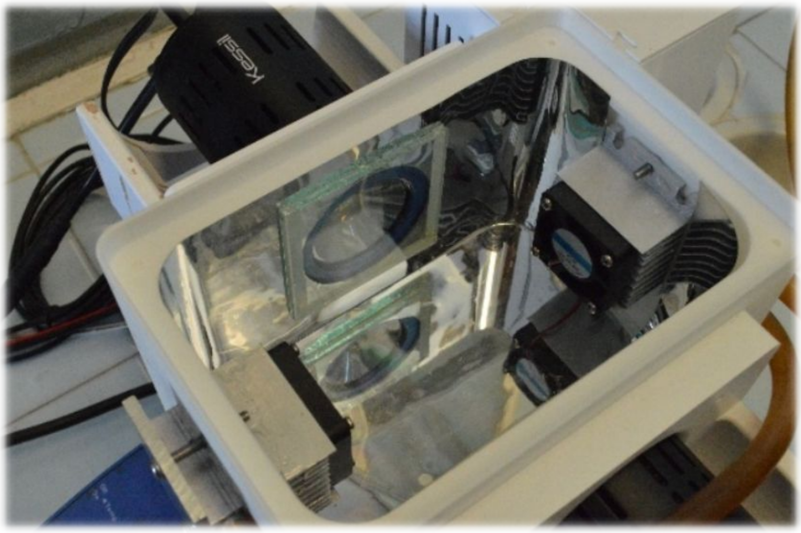

Figure S2. Picture of the inside of the reactor

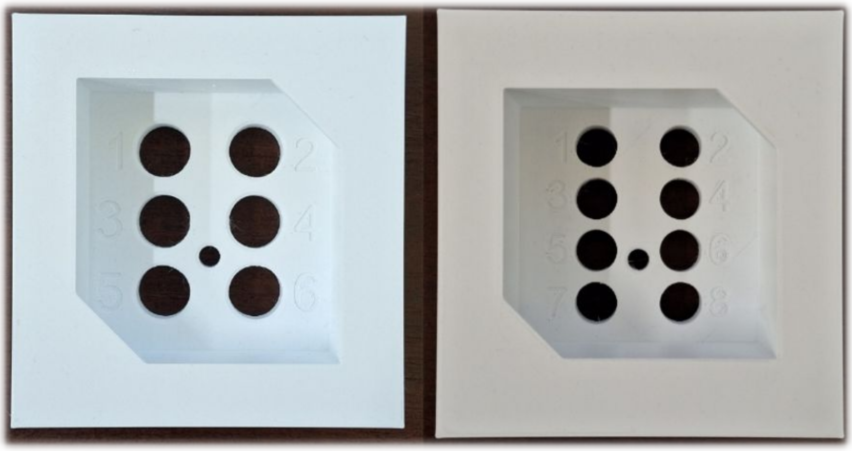

Figure S3. Picture of the 4 mL and 1.5 mL vial holders

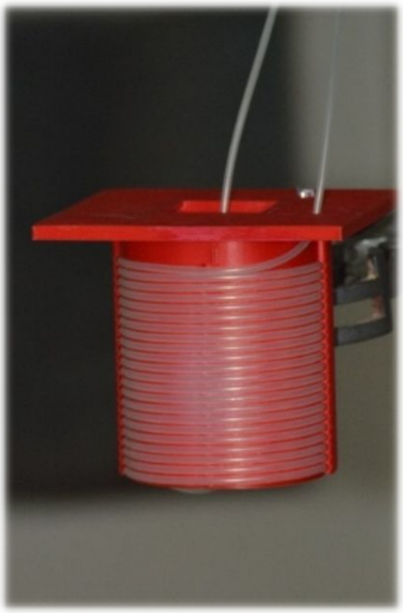

Figure S4. Picture of the flow insert

2. Characterization of new materials

2.1. Dibenzyl 2-(2,2-diphenylethyl)malonate (7a):

NMR spectrum in $CDCl_3$:

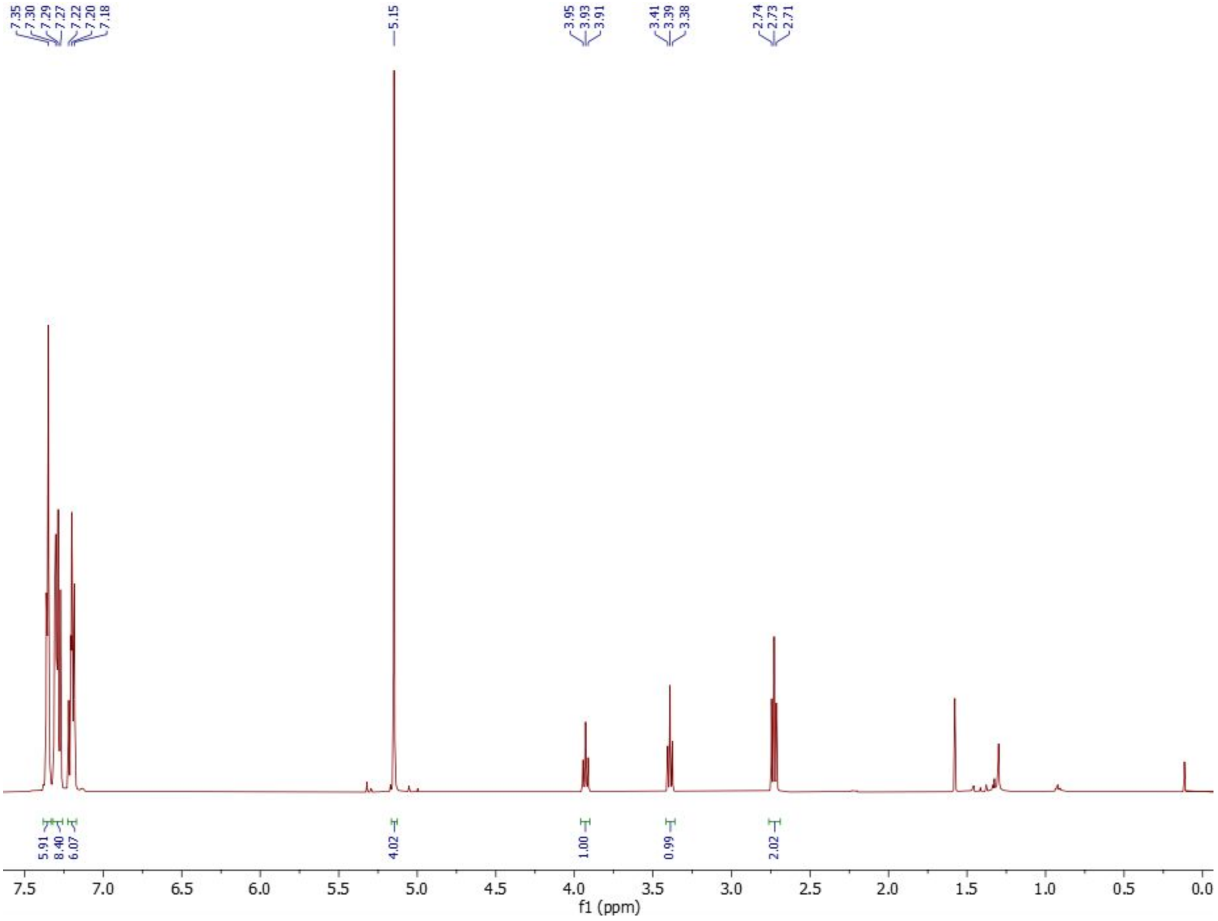

Figure S5. ^{1}H NMR spectrum of dibenzyl 2-(2,2-diphenylethyl)malonate (**7a**)

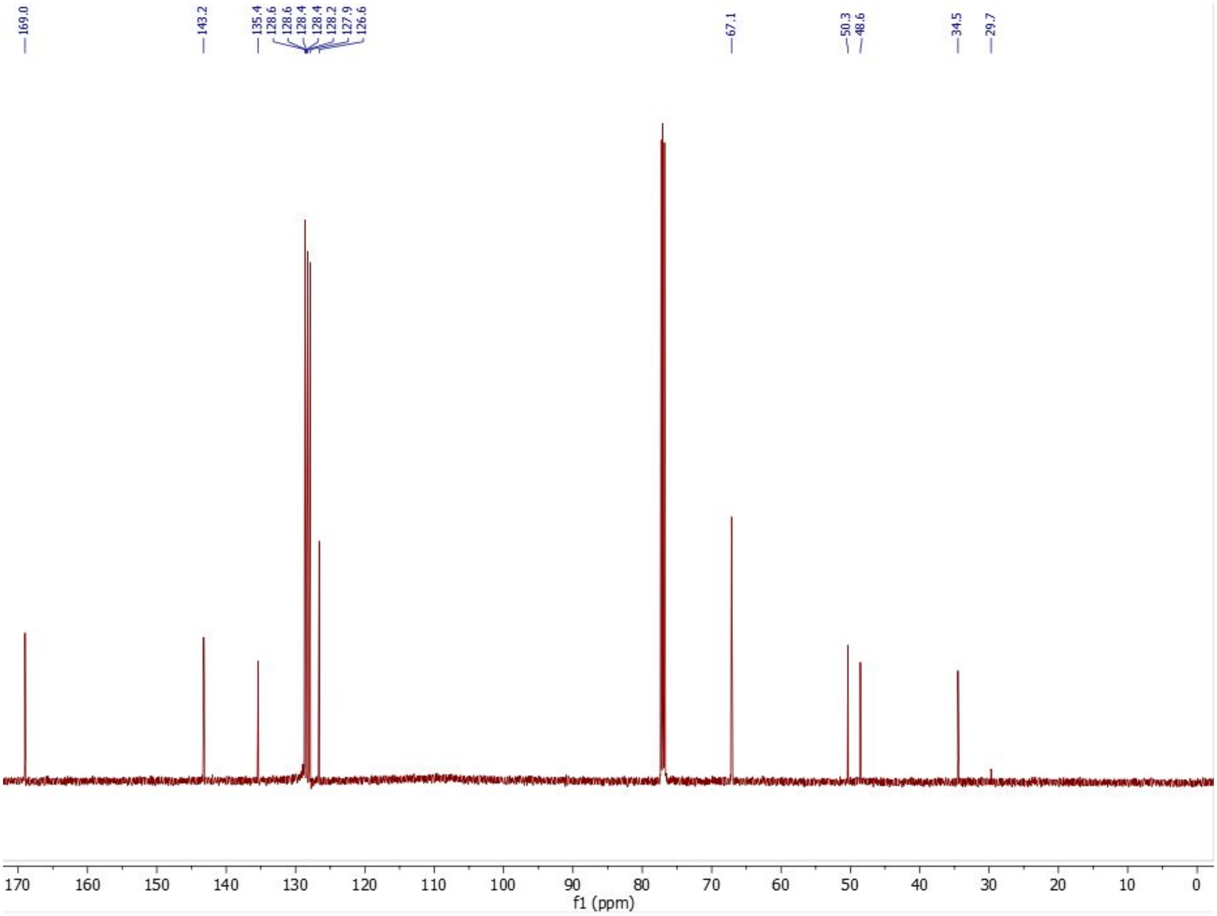

Figure S6. ^{13}C NMR spectrum of dibenzyl 2-(2,2-diphenylethyl)malonate (**7a**)

<u>HRMS spectrum:</u>

HRMS (ESI$^+$): *m/z* [M + Na]$^+$ calcd. for $C_{31}H_{28}O_4$: 487.1885; found: 487.1885.

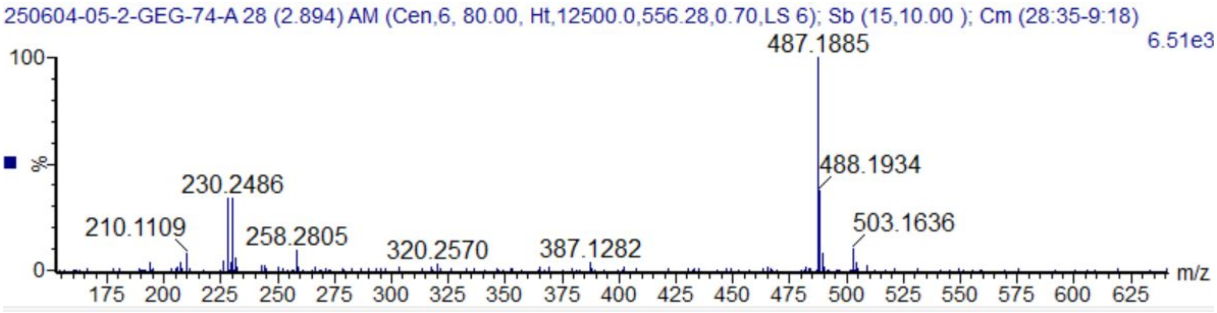

Figure S7. HRMS spectrum of dibenzyl 2-(2,2-diphenylethyl)malonate (**7a**)

2.2. 3,3-Diphenylheptane-2,6-dione (8b):

NMR spectrum in $CDCl_3$:

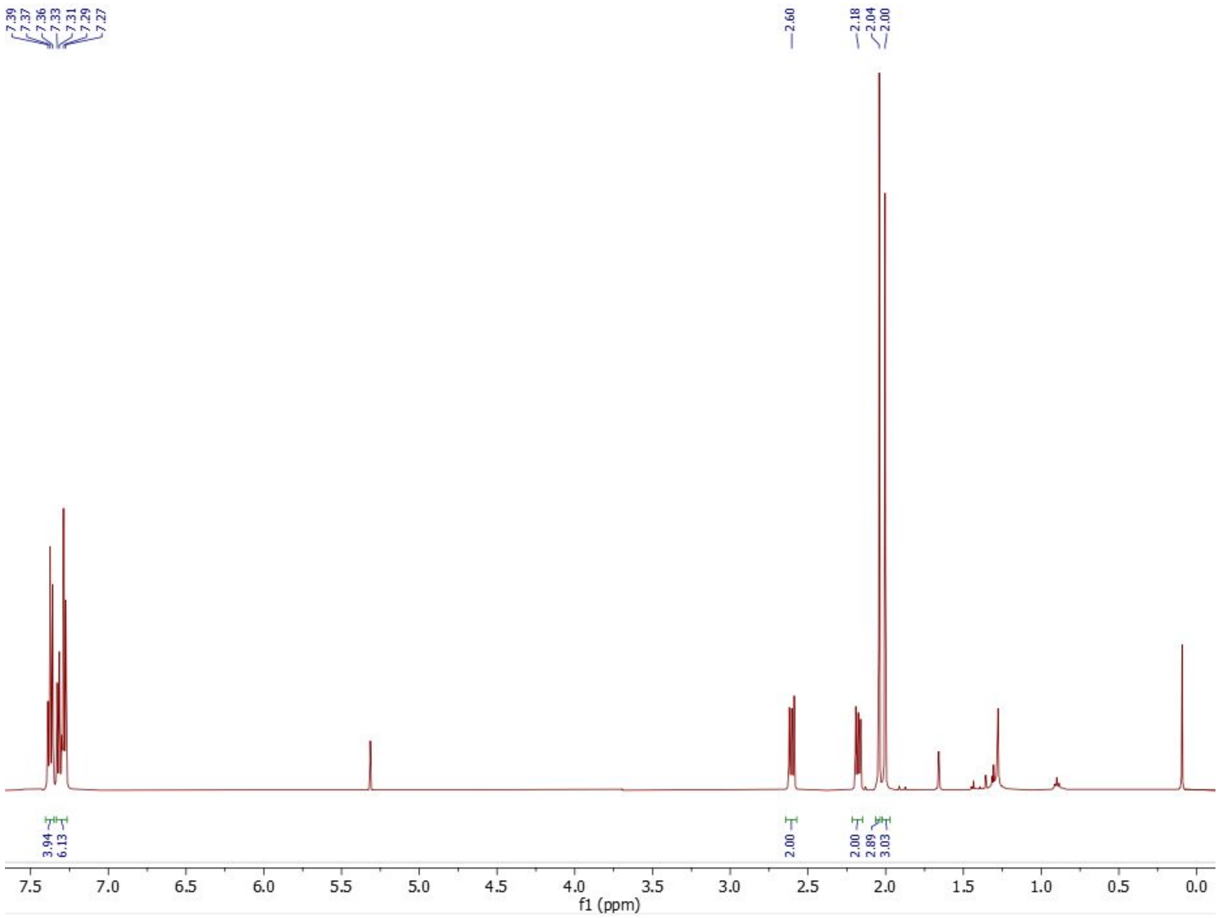

Figure S8. ^{1}H NMR spectrum of 3,3-diphenylheptane-2,6-dione (**8b**)

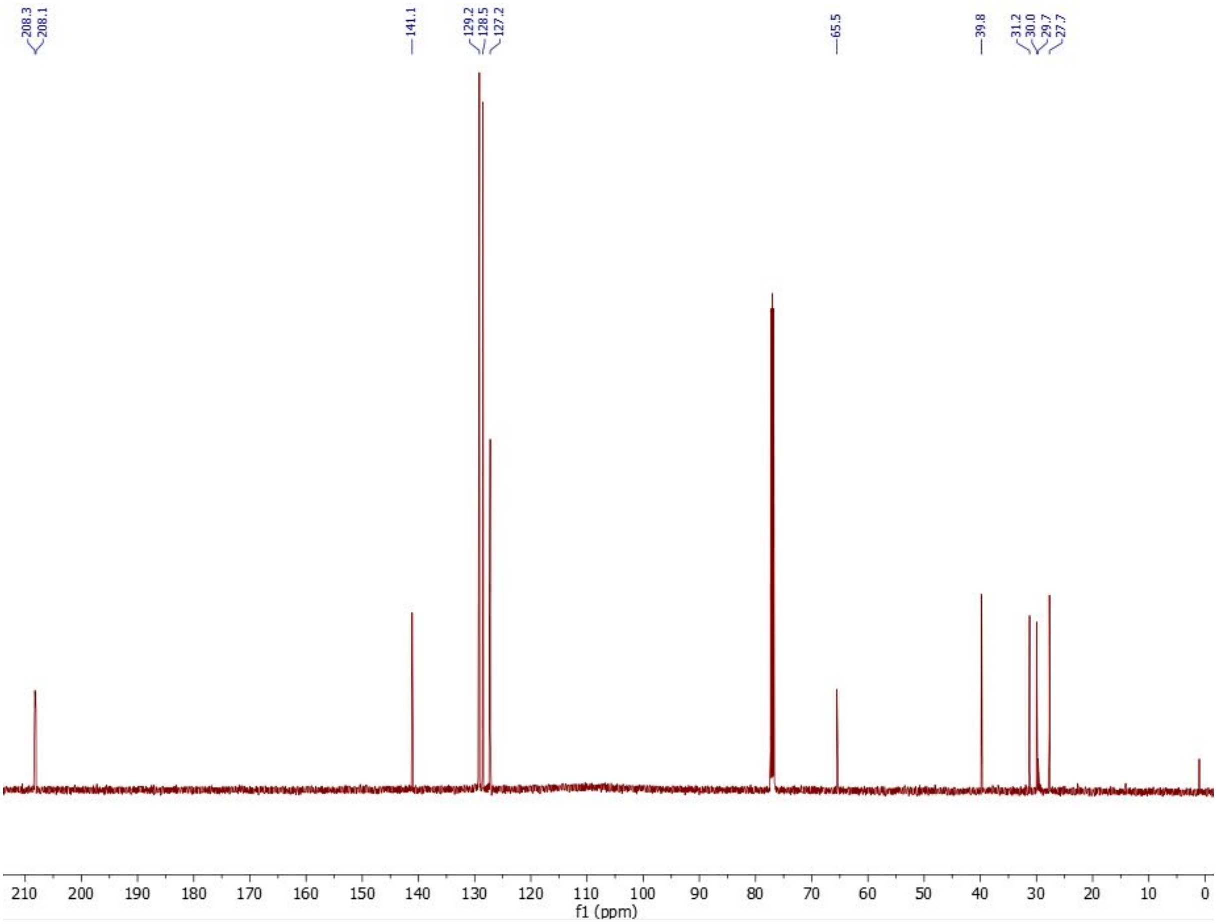

Figure S9. ^{13}C NMR spectrum of 3,3-diphenylheptane-2,6-dione (**8b**)

<u>HRMS spectrum:</u>

HRMS (ESI$^+$): *m/z* [M + Na]$^+$ calcd. for $C_{31}H_{28}O_4$: 303.1361; found: 303.1362.

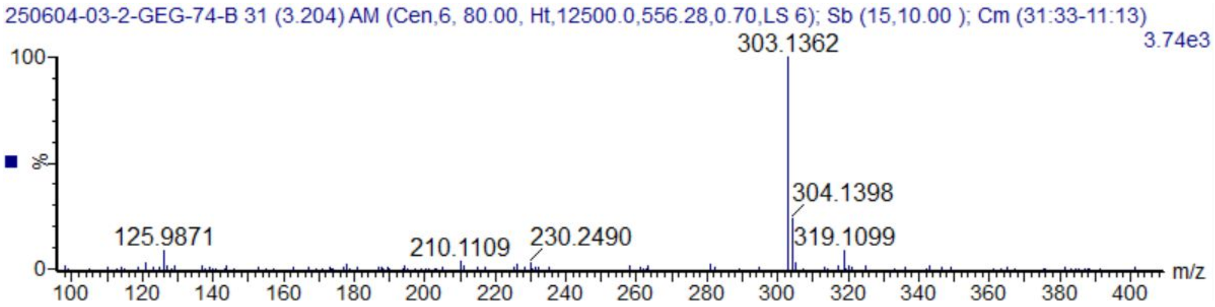

Figure S10. HRMS spectrum of of 3,3-diphenylheptane-2,6-dione (**8b**)

2.3. Ethyl 5-oxo-4,4-diphenylhexanoate:

NMR spectrum in $CDCl_3$:

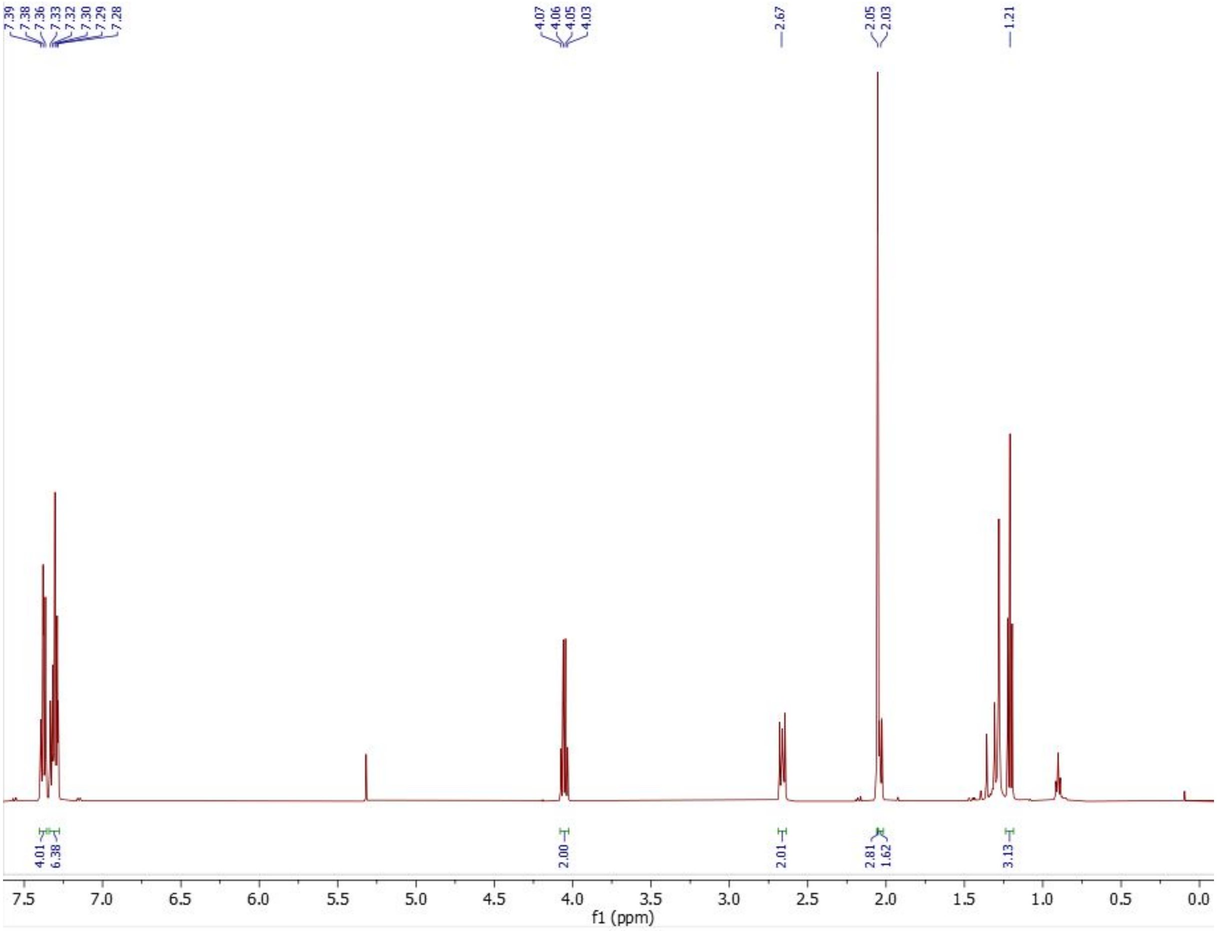

Figure S11. ^{1}H NMR spectrum of ethyl 5-oxo-4,4-diphenylhexanoate (**8c**)

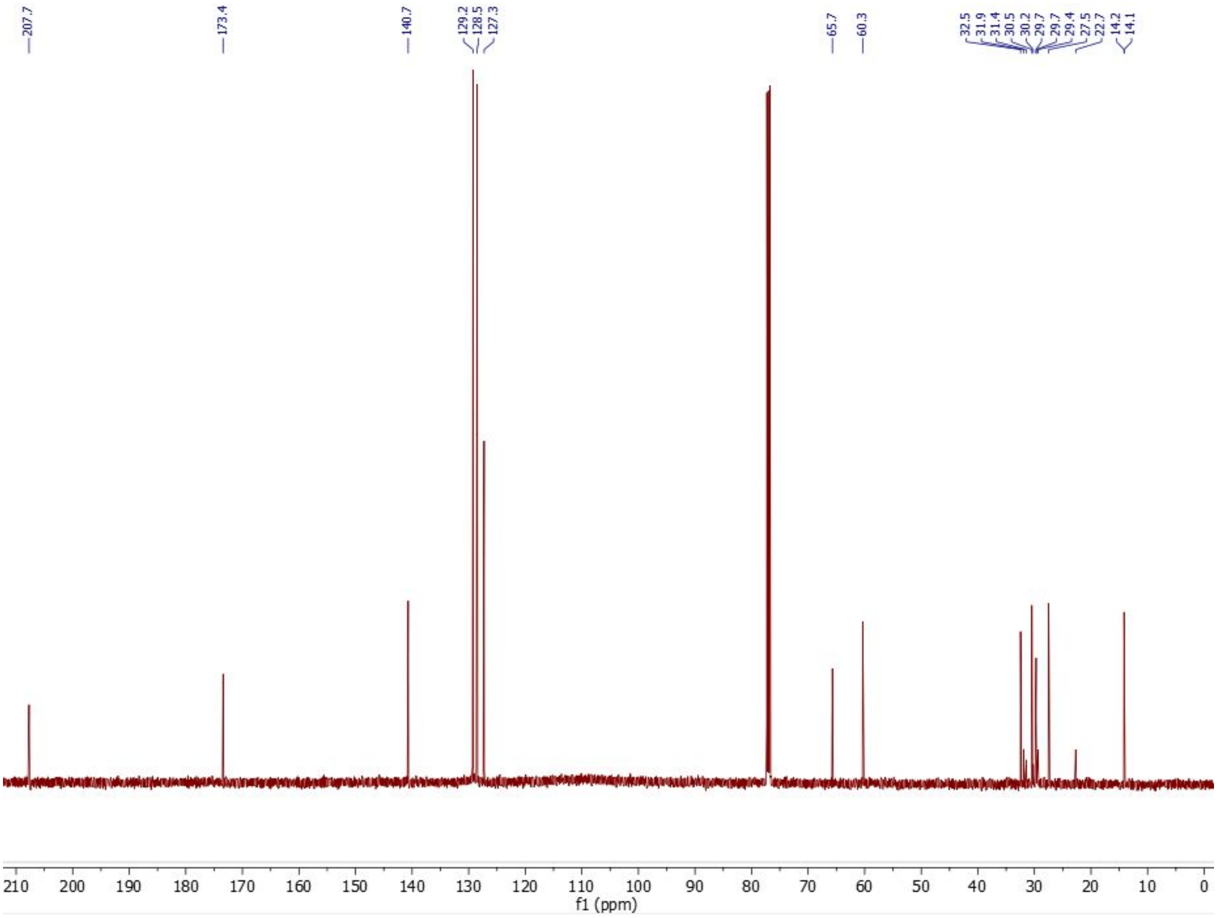

Figure S12. ^{13}C NMR spectrum of ethyl 5-oxo-4,4-diphenylhexanoate (**8c**)

<u>HRMS spectrum:</u>

HRMS (ESI^{+}): *m/z* [M + Na]$^{+}$ calcd. for $C_{31}H_{28}O_4$: 333.1467; found: 333.1470.

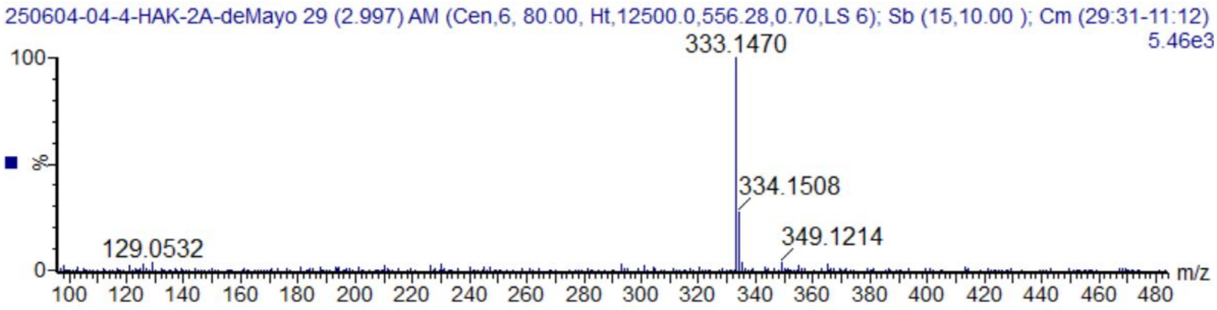

Figure S13. HRMS spectrum of ethyl 5-oxo-4,4-diphenylhexanoate (**8c**)

2.4. Methyl 2-cyano-4,4-diphenylbutanoate (7d):

NMR spectrum in $CDCl_3$:

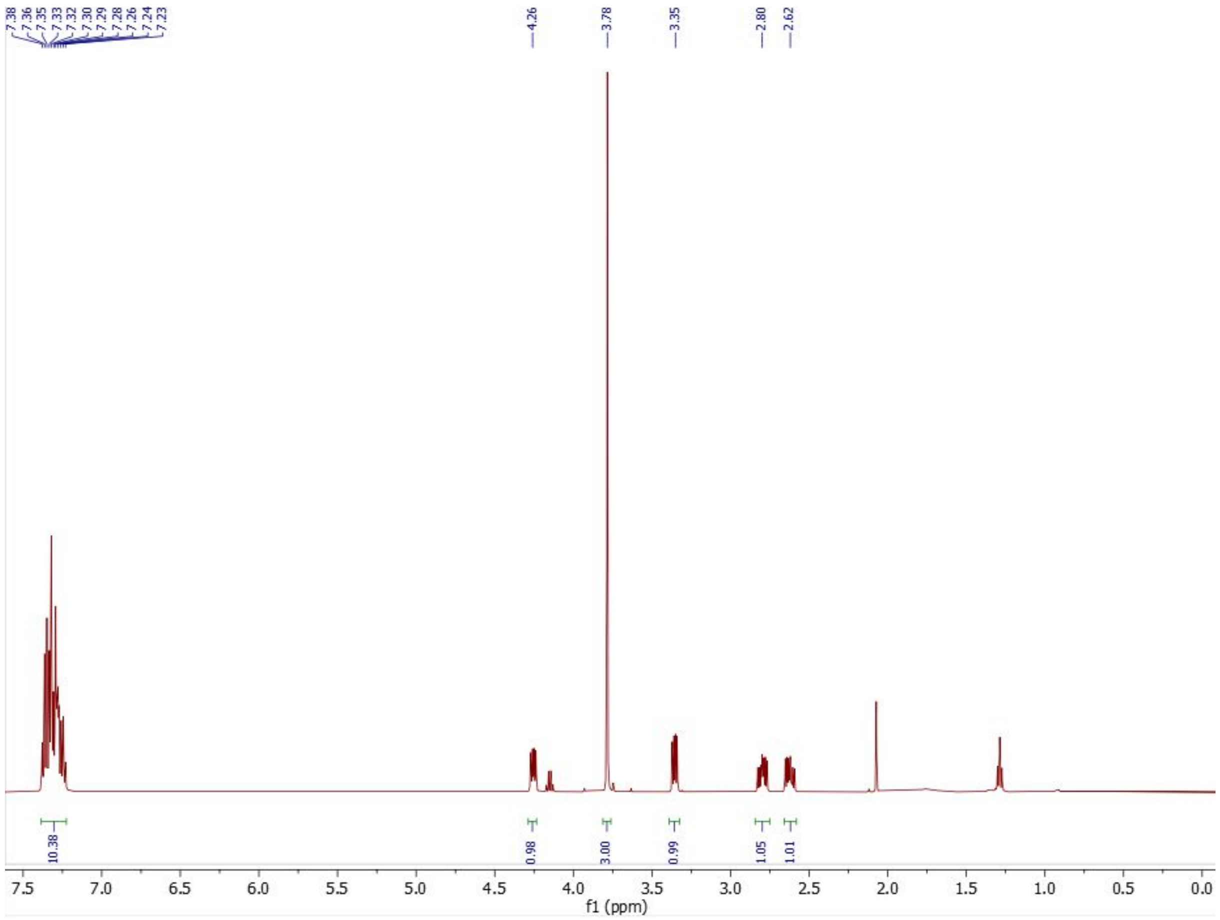

Figure S14. ^{1}H NMR spectrum of methyl 2-cyano-4,4-diphenylbutanoate (**7d**)

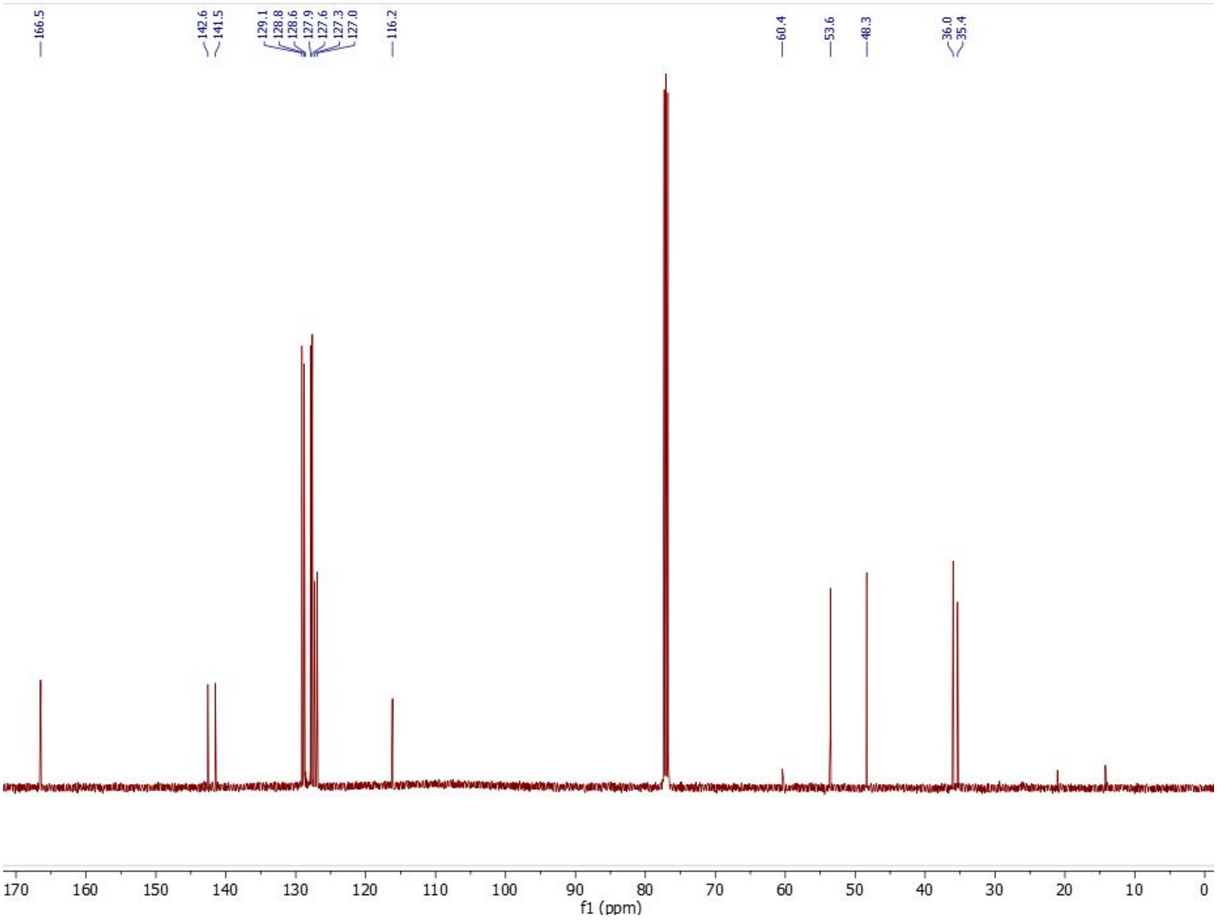

Figure S15. ^{13}C NMR spectrum of methyl 2-cyano-4,4-diphenylbutanoate (**7d**)

HRMS spectrum:

HRMS (ESI$^+$): *m/z* [M + Na]$^+$ calcd. for $C_{31}H_{28}O_4$: 302.1157; found: 302.1152.

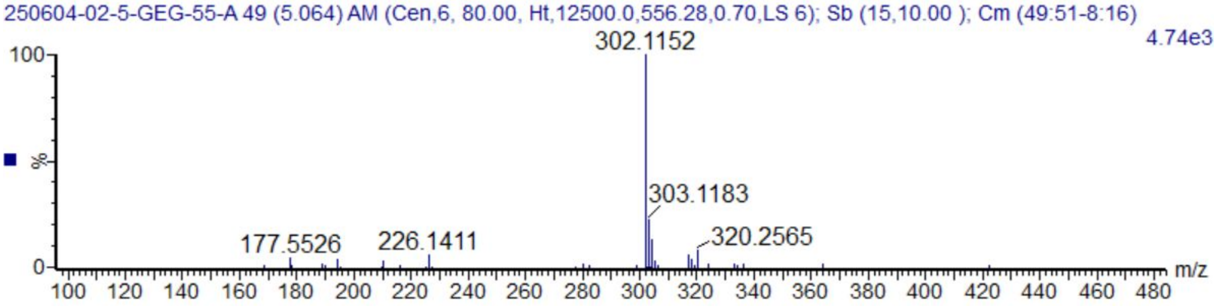

Figure S16. HRMS spectrum of methyl 2-cyano-4,4-diphenylbutanoate (**7d**)

3. HPLC yield determination for alkylated methyl cyanoacetate (7d)

HPLC method: HALO 90 Å C18 2.7 µm, 4.6×150 mm column, eluent CH_3CN/H_2O (0.1% formic acid) 5% to 95% CH_3CN in 11 min, then back to 5% CH_3CN in 5 min; 0.8 mL min^{-1}, column 40 °C; UV detector 230 nm, 35 °C. For the calibration curve, measurements were taken from pure reference product at 0.2, 0.4, 0.6, 0.8 and 1 mg mL^{-1} concentration.

4CzIPN:

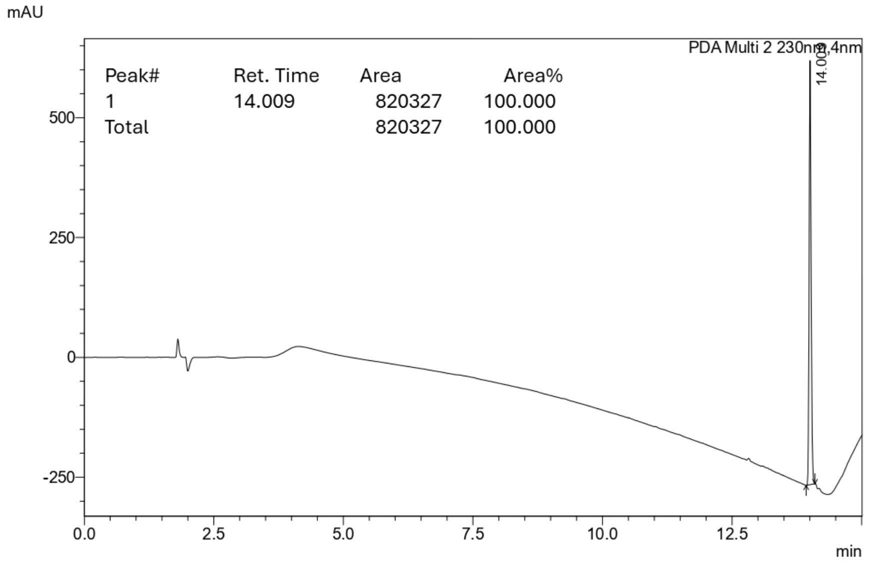

Figure S17. HPLC chromatogram of 4CzIPN

DBU:

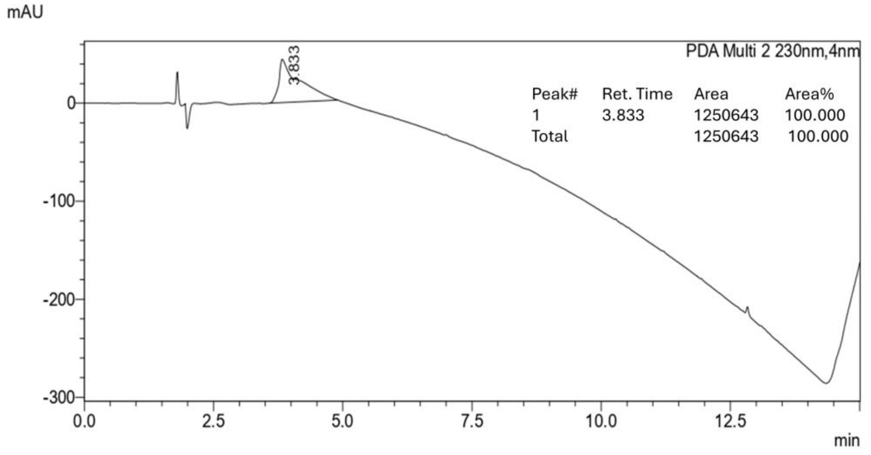

Figure S18. HPLC chromatogram of DBU

1,1-Diphenylethylene (**4**):

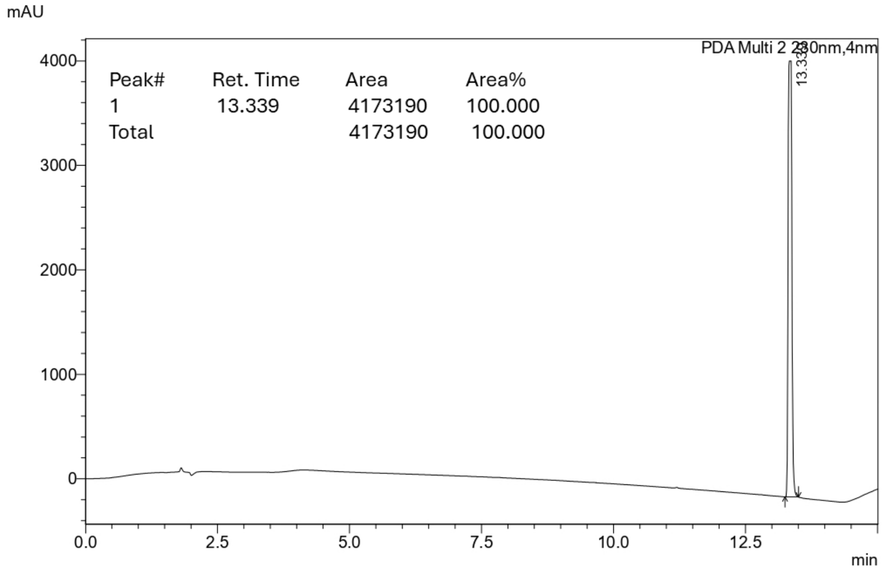

Figure S19. HPLC chromatogram of 1,1-diphenylethylene (**4**)

Methyl cyanoacetate (**6d**):

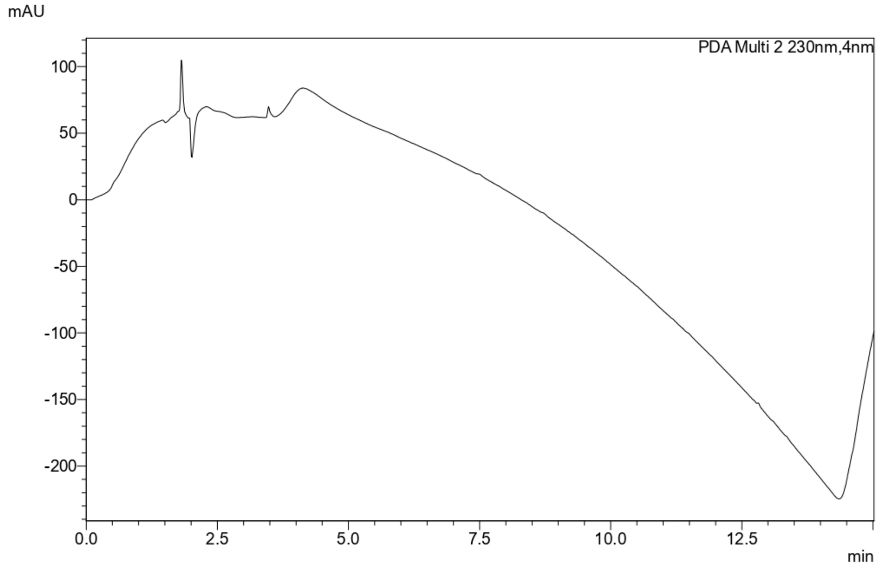

Figure S20. HPLC chromatogram of methyl cyanoacetate (**6d**) – cannot be seen at 230 nm

Alkylated methyl cyanoacetate (**7d**):

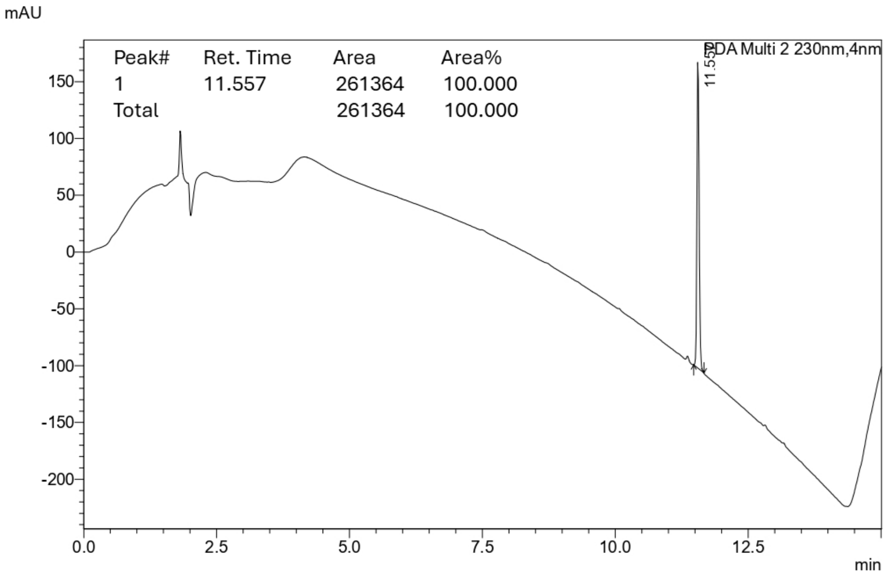

Figure S21. HPLC chromatogram of alkylated methyl cyanoacetate (**7d**)

Reaction mixture:

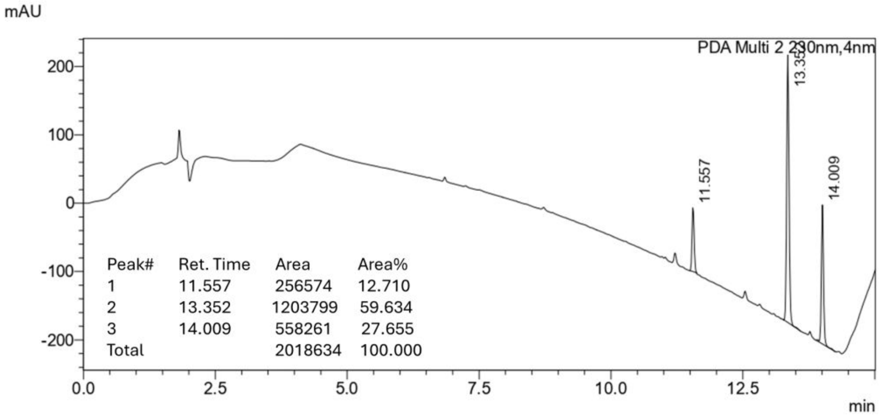

Figure S22. Example of a HPLC chromatogram of a reaction mixture

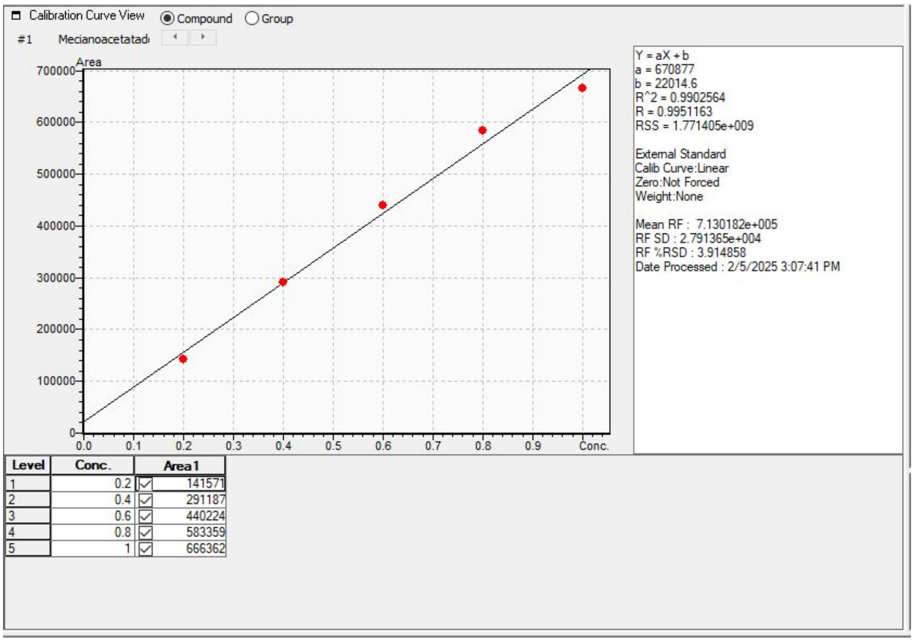

Figure S23. Calibration curve for determining the alkylated methyl cyanoacetate (**7d**) yields

4. Investigation of the potential effect of the reaction vial placement on the yields

Eight simultaneous reactions were carried out on 1.5 mL scale with methyl cyanoacetate to investigate the potential differences in the yields dependent on the placement in the reactor. Reaction conditions: MeCN solvent, 1 mol% 4CzIPN, 6 mol% DBU, 1.2 eq. methyl cyanoacetate, 20 °C, 6 hours. Based on Figure S18, it is visible that the placement does not affect the yields of the reaction; therefore, it can be neglected during the DoE process.

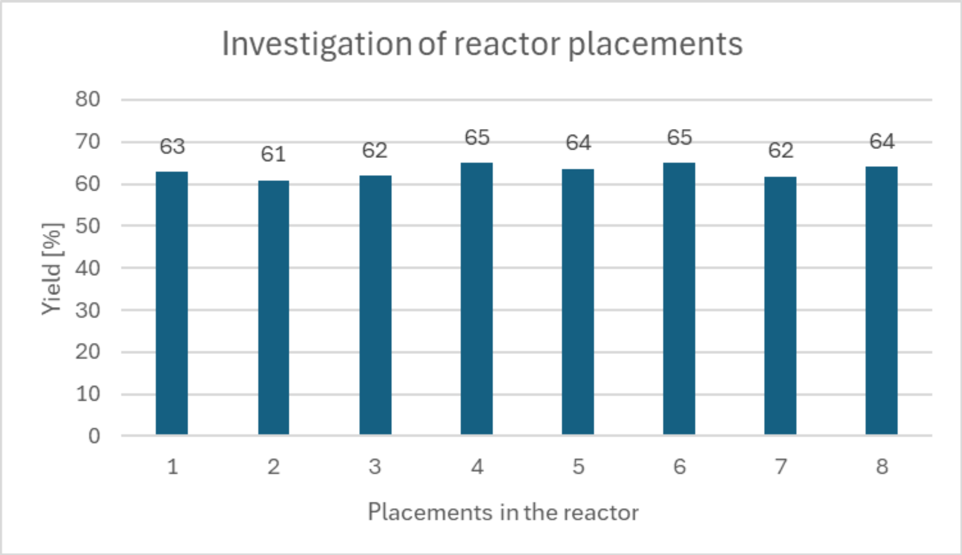

Figure S24. Results of the investigation of potential reactor placement effect on the yields

5. Preliminary studies for Design of Experiments

5.1. Base and catalyst amount screening

Preliminary studies have been conducted to determine the amount of base and catalyst to use during the DoE experiments. For the DBU, 1–10 mol% was chosen based on the results of a previous publication,[1] while the 4CzIPN amount was screened between 0.1–5 mol%. In both cases, a local maximum was visible in the 2–4 mol% range, which was determined to be ideal for conducting the design of experiments investigations. (Figure S19.)

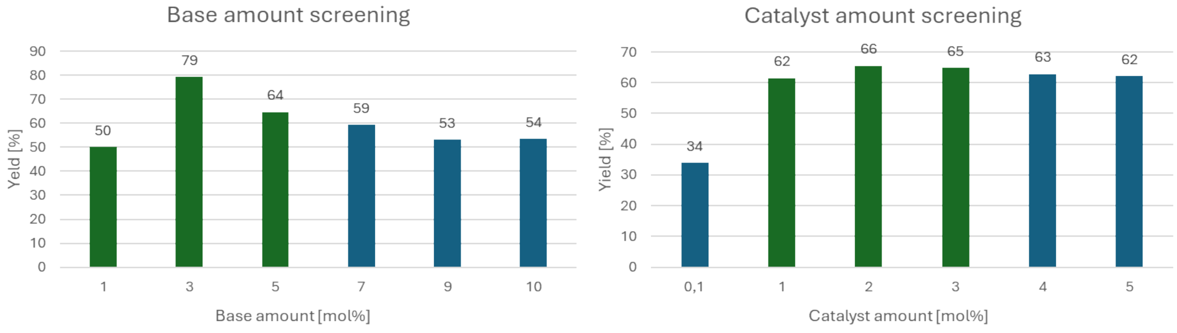

Figure S25. Results of the base and catalyst amount screening reactions

5.2. Investigation of the effect of light intensity on the C-C bond forming reaction

Preliminary studies have been conducted to determine the effect of the light intensity on the outcome of the reaction of methyl cyanoacetate (**6d**) and 1,1-diphenyethylene (**4**). The reactions were irradiated for 5 hours using 1 mol% 4CzIPN and 6 mol% DBU in acetonitrile. As Table S1 shows, the yield was only marginally lower at 50% intensity than at 100% intensity, and the reaction did not proceed in the natural light. Based on these preliminary experiments, the light intensity has no significant effect on the yield, therefore we set a medium value (75%) during our further experiments.

Table S1. Effect of the light intensity on the reaction yield between methyl cyanoacetate (**6d**) and 1,1-diphenyethylene (**4**)

Light intensity	Yield [%]
50%	60
75%	62
100%	59
natural light (over 3 days)	0

6. Further results of Design of experiments

6.1. Full effects estimate tables of the DoE models including confidence limits

Effect Estimates; Var.:Yiled (%); R-sqr=,99847; Adj:,99081 (ADATOK 2 in Kisterv 2.0)
2**(4-0) design; MS Residual=2,109699
DV: Yiled (%)

Factor	Effect	Std.Err.	t(3)	p	-95,% Cnf.Limt	+95,% Cnf.Limt	Coeff.	Std.Err. Coeff.	-95,% Cnf.Limt	+95,% Cnf.Limt
Mean/Interc.	60,1237	0,363120	165,5754	0,000000	58,9681	61,2794	60,1237	0,363120	58,9681	61,2794
Curvatr.	-3,0372	1,827664	-1,6618	0,195139	-8,8537	2,7792	-1,5186	0,913832	-4,4268	1,3896
(1)Temperature (°C)	-23,9947	0,726240	-33,0396	0,000061	-26,3059	-21,6835	-11,9973	0,363120	-13,1530	-10,8417
(2)Base (mol%)	2,8161	0,726240	3,8777	0,030371	0,5049	5,1273	1,4081	0,363120	0,2524	2,5637
(3)Catalyst (mol%)	-3,3576	0,726240	-4,6233	0,019051	-5,6688	-1,0464	-1,6788	0,363120	-2,8344	-0,5232
(4)Molar ratio (-)	19,0812	0,726240	26,2739	0,000121	16,7700	21,3924	9,5406	0,363120	8,3850	10,6962
1 by 2	4,6059	0,726240	6,3420	0,007929	2,2946	6,9171	2,3029	0,363120	1,1473	3,4585
1 by 3	2,4093	0,726240	3,3175	0,045137	0,0981	4,7206	1,2047	0,363120	0,0491	2,3603
1 by 4	0,2367	0,726240	0,3259	0,765920	-2,0745	2,5479	0,1183	0,363120	-1,0373	1,2739
2 by 3	3,9430	0,726240	5,4294	0,012262	1,6318	6,2543	1,9715	0,363120	0,8159	3,1271
2 by 4	-0,2063	0,726240	-0,2841	0,794804	-2,5176	2,1049	-0,1032	0,363120	-1,2588	1,0524
3 by 4	-1,0421	0,726240	-1,4350	0,246786	-3,3534	1,2691	-0,5211	0,363120	-1,6767	0,6345
1*2*3	-5,1605	0,726240	-7,1058	0,005735	-7,4717	-2,8493	-2,5802	0,363120	-3,7359	-1,4246
1*2*4	-0,2724	0,726240	-0,3751	0,732561	-2,5836	2,0388	-0,1362	0,363120	-1,2918	1,0194
1*3*4	-0,8425	0,726240	-1,1601	0,329945	-3,1537	1,4687	-0,4213	0,363120	-1,5769	0,7344
2*3*4	0,1252	0,726240	0,1725	0,874054	-2,1860	2,4365	0,0626	0,363120	-1,0930	1,2182

Figure S26. Full effects estimate table for the 2^4 DoE before reduction including confidence limits

Effect Estimates; Var.:Yiled (%); R-sqr=,91099; Adj:,89319 (ADATOK 2 in Kisterv 2.0)
2**(4-0) design; MS Residual=24,52473
DV: Yiled (%)

Factor	Effect	Std.Err.	t(15)	p	-95,% Cnf.Limt	+95,% Cnf.Limt	Coeff.	Std.Err. Coeff.	-95,% Cnf.Limt	+95,% Cnf.Limt
Mean/Interc.	60,1237	1,238061	48,56282	0,000000	57,4849	62,7626	60,1237	1,238061	57,4849	62,76261
Curvatr.	-3,0372	6,231439	-0,48740	0,633021	-16,3192	10,2448	-1,5186	3,115719	-8,1596	5,12239
(1)Temperature (°C)	-23,9947	2,476123	-9,69043	0,000000	-29,2724	-18,7170	-11,9973	1,238061	-14,6362	-9,35848
(4)Molar ratio (-)	19,0812	2,476123	7,70608	0,000001	13,8035	24,3589	9,5406	1,238061	6,9017	12,17946

Figure S27. Full effects estimate table for the reduced model including confidence limits

6.2. Checking the constant error variance assumption

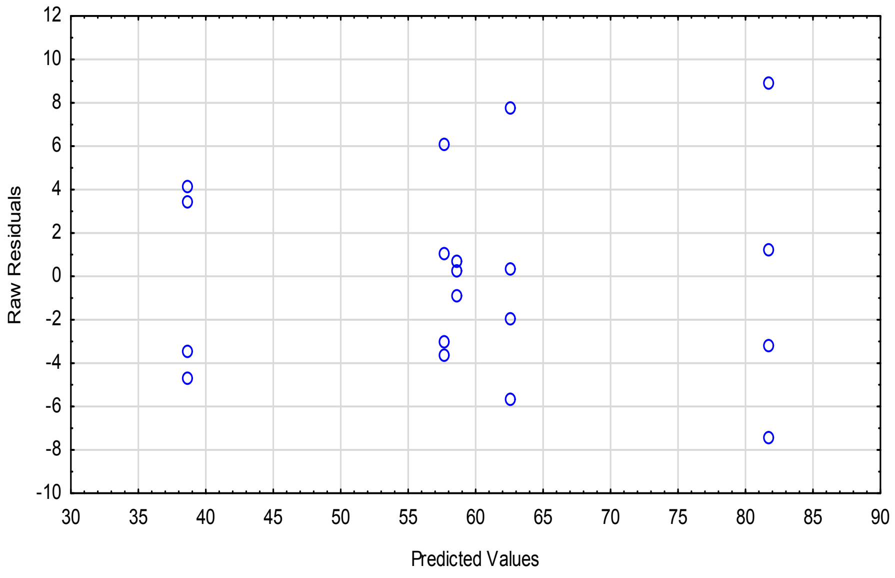

Figure S28. Predicted vs. residual values of alkylated methyl cyanoacetate (**7d**) yields

The residual values are in one area and do not show a particular pattern; therefore, constant error variance can be assumed.

6.3.Checking the normal distribution of errors assumption

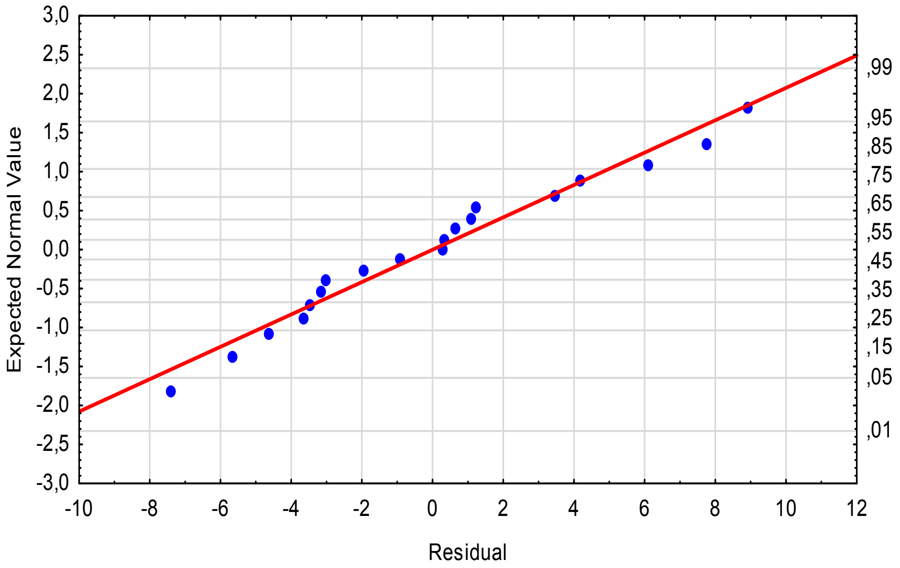

Figure S29. Gauss net of error distribution

The residuals follow along the diagonal well; therefore, a normal distribution of errors can be assumed.

References

(1) Baś, S.; Yamashita, Y.; Kobayashi, S. Development of Brønsted Base-Photocatalyst Hybrid Systems for Highly Efficient C-C Bond Formation Reactions of Malonates with Styrenes. *ACS Catal* **2020**, *10*, 10546–10550. https://doi.org/10.1021/acscatal.0c02716.
